# pH- and ligand-induced release of loads from DNA–acrylamide hydrogel microcapsules†
†Electronic supplementary information (ESI) available: Nucleic acid sequences; characterization of nucleic acid-modified copolymers; SEM images, confocal images of microcapsules; calibration curves of TMR-D, TR-D, and DOX-D; synthesis of DOX-D; CD spectra of pH-responsive hydrogel; characterization of hydrogel membranes. See DOI: 10.1039/c6sc04770j



**DOI:** 10.1039/c6sc04770j

**Published:** 2017-01-10

**Authors:** Wei-Ching Liao, Sivan Lilienthal, Jason S. Kahn, Marianna Riutin, Yang Sung Sohn, Rachel Nechushtai, Itamar Willner

**Affiliations:** a Institute of Chemistry , Center for Nanoscience and Nanotechnology , The Hebrew University of Jerusalem , Jerusalem 91904 , Israel . Email: willnea@vms.huji.ac.il; b Institute of Life Science , The Hebrew University of Jerusalem , Jerusalem 91904 , Israel

## Abstract

Herein, a method to construct stimuli-responsive DNA–acrylamide-based hydrogel microcapsules has been presented. This method involves the use of polyacrylamide chains modified with predesigned nucleic acid hairpin units and optionally single-strand tethers that provide the required hybridization and recognition functions to yield substrate-loaded stimuli-responsive hydrogel-based microcapsules. The synthesis of the microcapsules involves the loading of CaCO_3_ microparticles with the respective load substrates and the functionalization of the CaCO_3_ template particles with nucleic acid promoter units. In the presence of the hairpin-modified acrylamide chains, the promoter units induce the hybridization chain reaction (HCR), which leads to the formation of a hydrogel coating, which, after the dissociation of the CaCO_3_ cores, yields substrate-loaded stimuli-responsive hydrogel microcapsules. One of the microcapsule systems includes, in the hairpin-modified acrylamide constructs, and in the subsequent HCR-generated hydrogel shells, the caged sequences of anti-ATP or anti-cocaine aptamers. In the presence of ATP or cocaine, the duplex-caged aptamer sequences are separated *via* the formation of ATP- or cocaine–aptamer complexes, which results in the partial separation of the microcapsules and the release of the loads. The second type of microcapsule is cooperatively stabilized by bridges generated by HCR and pH-sensitive duplex units. Under acidic conditions, the pH-sensitive bridges dissociate *via* the formation of i-motif structures, which results in an increase in the fluidity of the microcapsule shells and the release of the loads. Preliminary studies indicate that ATP- or pH-responsive microcapsules loaded with the anticancer drug, doxorubicin, have a selective cytotoxic effect on MDA-MB-231 cancer cells.

## Abstract 2

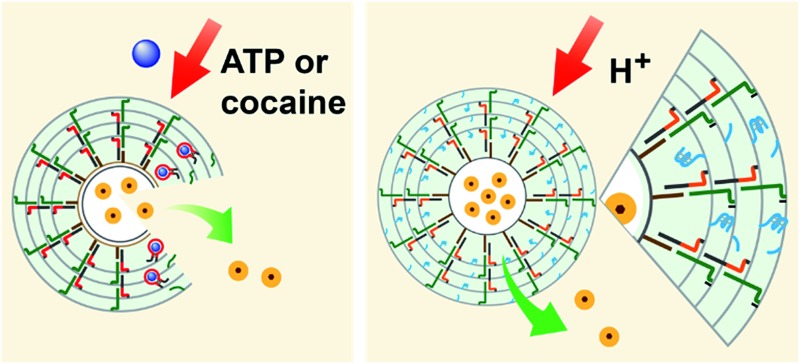
A generic method of preparing stimuli-responsive substrate-loaded hydrogel microcapsules, composed of polyacrylamide chains cross-linked by nucleic acids, has been described. The triggered release of loads from the microcapsules proceeds *via* either the formation of an ATP aptamer or a cocaine aptamer, or the pH-induced generation of i-motif structures.

## Introduction

The preparation of substrate-loaded microcapsules has attracted significant research efforts during the past decade,^1^ and different applications of the loaded microcapsules, such as drug carriers,^2^ sensors,^3^ imaging agents,^4^ and microreactors,^5^ have been suggested. Methods for preparing the microcapsules included the electrostatically driven layer-by-layer deposition of charged polymers on the loaded microparticle cores,^6^ the assembly of functional polymer layers on the loaded microparticle cores by supramolecular host–guest interactions,^7^ or the formation of chemical bonds, *e.g.*, disulfide bonds,^8^ followed by the dissolution of the core particles. The synthesis of stimuli-responsive or stimuli-degradable loaded microcapsules is particularly interesting for controlled drug release applications.^9^ Different triggers, such as heat,^10^ light,^11^ pH,^12^ magnetic fields,^13^ and chemical and biochemical reactions,^14^ have been used to degrade the microcapsules and release the loads. Similarly, the preparation of hydrogel-stabilized loaded microcapsules has been reported, and the degradation of the hydrogel boundary was suggested as a mechanism for releasing the loads.^15^

DNA-stabilized-loaded microcapsules, in particular stimuli-responsive DNA-based microcapsules, have attracted recent research efforts.^9^ The base complementarity of nucleic acids provides a general mechanism for constructing layers on the loaded core template microparticles, and the subsequent dissolution of the template cores provides a general method of assembling the loaded microcapsules. Furthermore, by the integration of stimuli-responsive nucleic acid units into the DNA shells of the microcapsules, signal-triggered degradable microcapsules have been designed. For example, photodegradable nucleic acids,^11^ pH-responsive nucleic acids (i-motifs, triplexes),^11^ and ligand–aptamer complexes^16,17^ have been used to degrade all-DNA-based microcapsules and to release the loads. The all-DNA-stabilized microcapsules suffer, however, from several limitations, which include the complex layer-by-layer deposition (*via* hybridization) of the functional nucleic acid layers, the single-cycle triggered unlocking of the microcapsules, which prevents switchable dose-controlled release of the loads, the sensitivity of the microcapsules to heat, the susceptibility of the microcapsules to enzymatic digestion, *e.g.*, by DNase or endonucleases, and the cost involved in the synthesis of the microcapsules.

The synthesis of stimuli-responsive DNA–acrylamide copolymer hydrogels is a rapidly developing research area.^18^ Different triggers such as pH,^19^ metal ions/ligands,^20^ K^+^/crown ethers,^21^ and light^11^ have been used to stimulate hydrogel-to-solution transitions. Recently, a method of assembling thin films of stimuli-responsive hydrogels on surfaces was developed.^22^ According to this method, two different types of acrylamide copolymer chains were modified with the DNA hairpins, H_A_ and H_B_, respectively, and also with nucleic acid tethers that correspond to subunits of the G-quadruplex. In the presence of a promoter strand associated with the surface, the hybridization chain reaction (HCR) was initiated between the two hairpin-functionalized chains,^23^ which led to crosslinking of the polymer chains by the nucleic acid duplexes and the formation of a hydrogel film on the surface. In the presence of K^+^ ions, the G-rich tethers formed G-quadruplex bridges as cooperative cross-linkers of the hydrogel. Exposure of the resulting hydrogel to 18-crown-6 ether led to the separation of the G-quadruplex bridging units. By this method and in the presence of K^+^ ions/crown ethers, the stiffness of the hydrogel film could be cycled between high and low values. In the present study, we report on the novel synthesis of stimuli-responsive DNA-based hydrogel microcapsules for the triggered release of loads trapped in the microcapsules. We follow a systematic characterization of different microcapsules and discuss their applications as drug release vehicles. The study is constructed in a modular fashion. In the first step, we addressed the development of a general concept for generating stimuli-responsive DNA-based hydrogel microcapsules based on the hybridization chain reaction (HCR). In this context, we demonstrated the loading of fluorescent dyes, namely, tetramethylrhodamine-modified dextran (TMR-D) or Texas Red-modified dextran (TR-D), in hydrogel microcapsules and the triggered release of the loads by transforming the hydrogel shells into a quasi-liquid state *via* the formation of ligand/aptamer complexes or pH-controlled i-motif structures. We demonstrate the versatility of the unlocking of the microcapsules by ligand/aptamer complexes by employing two different ligands, namely, cocaine and adenosine triphosphate (ATP), to release the fluorescent loads. The “tool-box” provided by the different stimuli for unlocking the hydrogel microcapsules and releasing the loads was then used to develop microcapsules carrying an anticancer drug, which are unlocked by the ATP ligand, which is overexpressed in cancer cells, or by acidic pH triggers present in cancer cells. Specifically, we loaded the anticancer drug doxorubicin (DOX) in ATP- or pH-responsive microcapsules and characterized the triggered release of the drug from the respective microcapsules. Finally, the doxorubicin-loaded ATP- and pH-responsive microcapsules were introduced into MDA-MB-231 cancer cells, and their cytotoxicity towards the cancer cells was characterized.

## Results and discussion

**Fig. 1:**
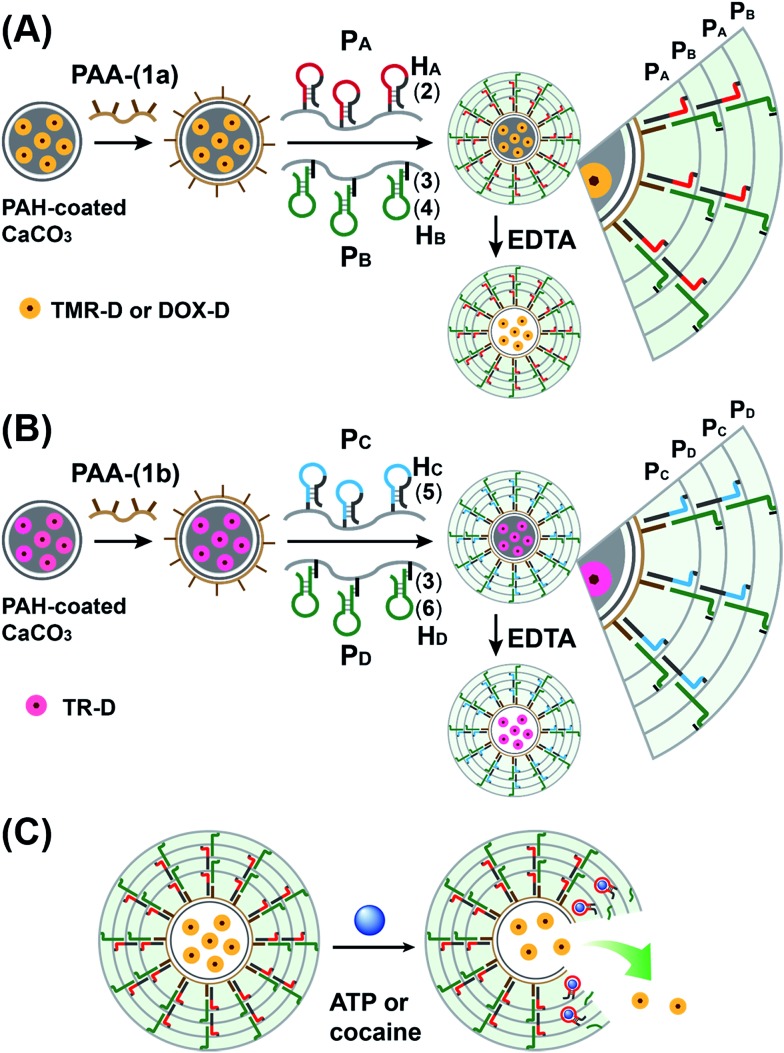
Schematic of aptamer–ligand responsive nucleic acid/acrylamide hydrogels *via* the promoter-induced hybridization chain reaction (HCR), which crosslinks hairpin-modified acrylamide chains. (A) Preparation of ATP-responsive hydrogel microcapsules. (B) Preparation of cocaine-responsive hydrogel microcapsules. (C) Schematic of the ligand-driven unlocking of the aptamer-bridged hydrogel microcapsules.

The preparation of the substrate-loaded aptamer–ligand stimuli-responsive hydrogel microcapsules is depicted in Fig. 1. CaCO_3_ microcapsules (3.5 μm) loaded with tetramethylrhodamine-modified dextran (TMR-D), Texas Red-modified dextran (TR-D), or doxorubicin-modified dextran (DOX-D) were coated with a positively charged layer of poly(allylamine hydrochloride) (PAH), and subsequently, a layer of negatively charged polyacrylic acid (PAA) was deposited on the positively charged primer polymer layer associated with the particles. An amine-modified nucleic acid (**1a**) or (**1b**), which acted as a promoter for the assembly of the microcapsule shells, was covalently linked to the PAA layer. Acrylamide copolymer chains P_A_ and P_B_ or P_C_ and P_D_ were used to prepare ATP- or cocaine-responsive hydrogel microcapsules. The polymer chains P_A_ and P_B_ were functionalized with the DNA hairpin H_A_ (**2**) and the hairpin conjugate H_B_ (**3**)/(**4**), respectively. Similarly, the copolymer chains P_C_ and P_D_ were bound to the hairpin H_C_ (**5**) and the hairpin conjugate H_D_ (**3**)/(**6**), respectively. Hairpin H_A_ in polymer chain P_A_ included an anti-ATP aptamer sequence in a caged configuration (marked in red). The promoter (**1a**) opened hairpin H_A_ (**2**) in a process that uncaged the aptamer sequence and yielded a toehold tether, which opened hairpin H_B_ associated with P_B_. The opening of hairpin H_B_ yielded a duplex between (**2**) and (**4**), which led to the crosslinking of P_A_ and P_B_. The opening of hairpin H_B_ yielded, however, a single-stranded domain that opened hairpin H_A_ and thus, induced the hybridization chain reaction (HCR), which led to the cross-opening of the hairpins and the formation of a crosslinked acrylamide hydrogel shell, consisting of the acrylamide chains P_A_ and P_B_ bridged by the (**2**)/(**3**)–(**4**) units. Similarly, the polymer chains P_C_ and P_D_ were used to construct cocaine-responsive microcapsules. Hairpin H_C_ (**5**) in P_C_ included an anti-cocaine aptamer sequence (blue) in a caged configuration. The promoter (**1b**) opened hairpin H_C_ (**5**) in a process that uncaged the aptamer sequence and yielded a toehold tether, which opened hairpin H_D_ associated with P_D_. The opening of hairpin H_D_ yielded a duplex between (**5**) and (**6**), which led to the crosslinking of P_C_ and P_D_. The opening of hairpin H_D_ yielded, however, a single-stranded domain that opened hairpin H_C_, and thus, led to the hybridization chain reaction (HCR), which led to the cross-opening of the hairpins and the formation of a crosslinked acrylamide hydrogel shell consisting of the acrylamide chains P_C_ and P_D_ bridged by the (**5**)/(**3**)–(**6**) units. The subsequent dissolution of the CaCO_3_ core templates with EDTA yielded microcapsules loaded with TMR-D, TR-D or DOX-D. The treatment of the resulting microcapsules with ATP or cocaine resulted in the dissociation of the duplexes (**2**)/(**4**) or (**5**)/(**6**) *via* the formation of the respective ATP–aptamer or cocaine–aptamer complexes. The separation of the duplexes (**2**)/(**4**) or (**5**)/(**6**) led to the partial separation of the shells of the hydrogel microcapsules, which allowed the diffusional release of the loads from the microcapsules (Fig. 1(C)).

**Fig. 2:**
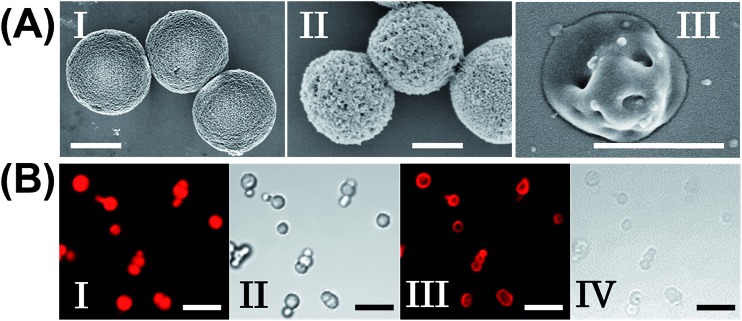
(A) SEM images corresponding to (I) uncoated CaCO_3_ particles loaded with TMR-D, (II) ATP-responsive hydrogel-coated CaCO_3_ particles, and (III) ATP-responsive hydrogel microcapsules after the dissolution of the CaCO_3_ core. Scale bars: 2 μm (panels I and II) and 1 μm (panel III). (B) Fluorescence confocal microscopy images and bright-field microscopy images corresponding to the microcapsules loaded with TMR-D before (I, II) and after (III, IV) the dissolution of the cores. Scale bars: 10 μm.

The molecular weights of polymer chains P_A_, P_B_, P_C_, and P_D_ were found by diffusion-ordered ^1^H NMR spectroscopy (DOSY) to be 145 kDa, 324 kDa, 670 kDa and 325 kDa, respectively. The ratios of acrylamide units to nucleic acid tethers were determined spectroscopically to be 423, 366, 100, and 366, respectively (see ESI Fig. S1–4†). Fig. 2(A) shows SEM images of the CaCO_3_ microparticles prior to their modification (panel I), after modification of the microparticles with the ATP-responsive hydrogel shell (panel II), and after the dissolution of the CaCO_3_ core by EDTA (panel III). We observe that the smooth CaCO_3_ particles are coated with a rough DNA-crosslinked polyacrylamide layer. The size of the coated microcapsules corresponds to ∼3.5 ± 0.8 μm. After the dissolution of the CaCO_3_ core, the microcapsules displayed a “flexible” amorphous structure (panel III). Fig. 2(B) shows confocal microscopy and bright-field microscopy images that correspond to the microcapsules loaded with TMR-D before and after the dissolution of the cores. The microcapsules with coated cores are strongly fluorescent (panel I), and the bright-field image (panel II) shows the solid cores coated with a thin film of the hydrogel. After the dissolution of the cores, the microcapsules display the fluorescence of the TMR-D load (panel III), and the bright-field image (panel IV) shows transparent microcapsules stabilized by the hydrogel shell. The microcapsules after the dissolution of the cores exhibit a smaller diameter (*ca.* 2.5 μm) in comparison to the microcapsules with coated cores (*ca.* 3.5 μm), which presumably originates from the shrinkage due to osmotic pressure of the microcapsules upon the dissolution of the cores.

**Fig. 3:**
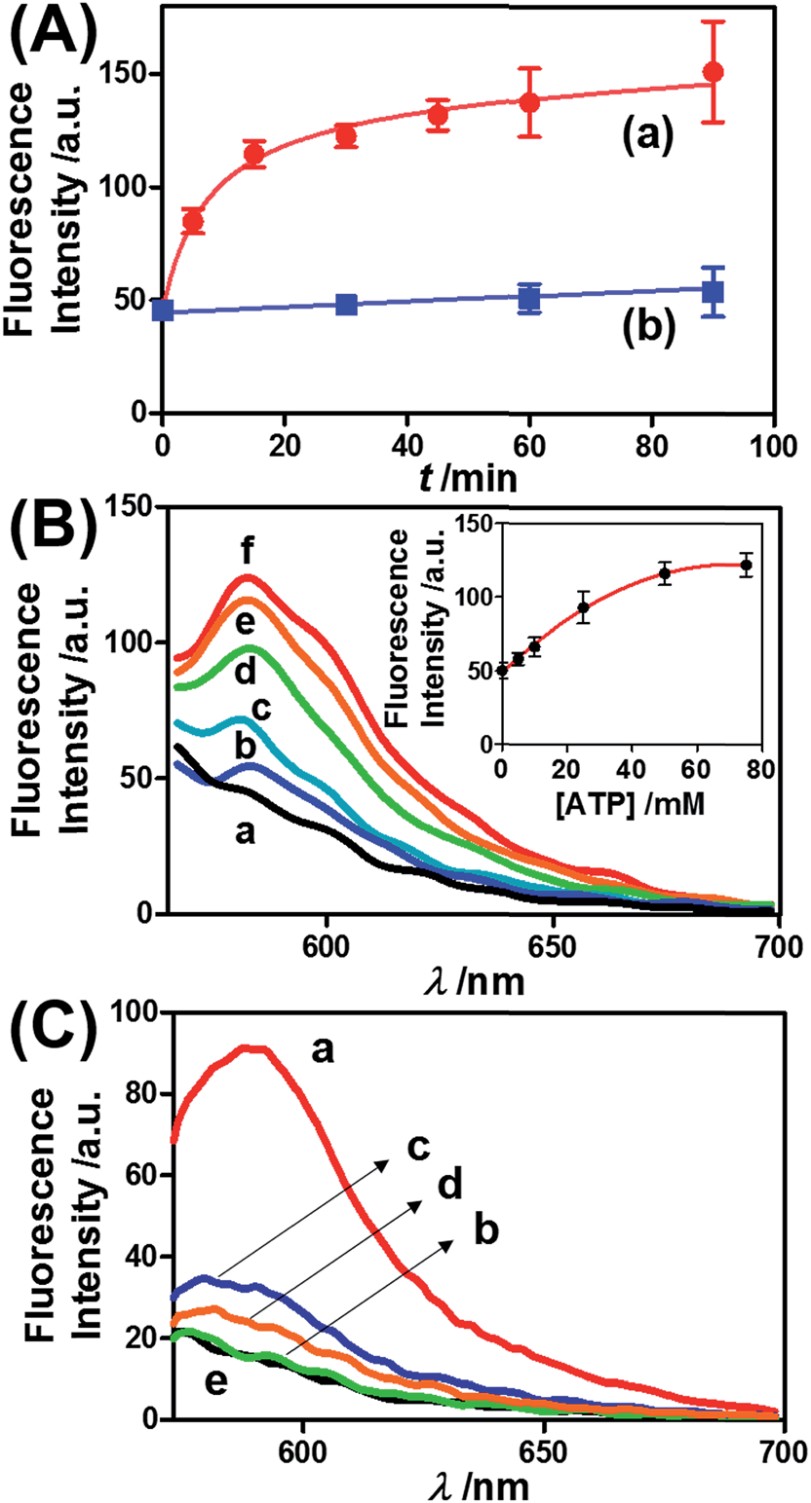
(A) Time-dependent release of TMR-D from the ATP-responsive hydrogel microcapsules treated with 50 mM ATP (a) and without added ATP (b). (B) Fluorescence spectra corresponding to the release of TMR-D upon exposing the microcapsules loaded with TMR-D to various concentrations of ATP for a fixed time interval of 30 min: (a) 0, (b) 5, (c) 10, (d) 25, (e) 50 and (f) 75 mM. Inset: derived calibration curve corresponding to the fluorescence intensities of the released TMR-D at *λ*_em_ = 582 nm upon treatment with different concentrations of ATP. (C) Fluorescence spectra corresponding to the release of TMR-D upon the treatment of the ATP-responsive microcapsules with different nucleotide triphosphates for a fixed time interval of 30 min: (a) 25 mM ATP, (b) 25 mM TTP, (c) 25 mM CTP, (d) 25 mM GTP, and (e) untreated.

Fig. 3 presents the ATP-driven release of TMR-D from the ATP-responsive acrylamide hydrogel microcapsules crosslinked by (**2**)/(**3**)–(**4**). No release of TMR-D from the capsules was detected in the absence of ATP. The exposure of the microcapsules to ATP (50 mM) resulted in the formation of an ATP–aptamer complex and the release of the TMR-D load. Fig. 3(A) depicts the time-dependent changes in the fluorescence of the bulk solution upon treatment of the microcapsules with ATP (50 mM). In these experiments, the ATP-treated microcapsules were precipitated at different time intervals, and the fluorescence of the supernatant solution was recorded. The fluorescence of the released load increased with time, and after *ca.* 30 minutes it leveled off at a constant value, which corresponded to the almost complete release of all the loaded TMR-D from the microcapsules. By knowing the concentration of the microcapsules from flow cytometry and using an appropriate calibration curve, we estimated that the average loading of TMR-D in a microcapsule was 5.0 × 10^–14^ mol. Fig. 3(B) depicts the release of TMR-D from the microcapsules as a function of the concentration of ATP. In these experiments, microcapsules loaded with a constant concentration of TMR-D were treated with variable concentrations of ATP for a fixed time interval of 30 minutes. As the concentration of ATP increased, the fluorescence intensity of the released TMR-D increased, which was consistent with the enhanced dissociation of the microcapsules as the ATP concentration increased. The ATP-driven release of the load was selective, and the treatment of the microcapsules with GTP, CTP, or TTP did not unlock the microcapsules (Fig. 3(C)). Thus, the selective formation of the ATP–aptamer complex led to the unlocking of the microcapsules and the release of TMR-D. It should be noted that the microcapsules remained with an intact structure and, after the release of TMR-D, the fluorescence of the microcapsules was depleted, but bright-field microscopy experiments indicated intact structures of the microcapsules. We assume that the formation of ATP–aptamer complexes in the hydrogel shells yielded pores or fluidic domains in the hydrogel shells that enabled the permeation/diffusion of the load through the shell (for further experiments that tested the stiffness of the hydrogel film before and after treatment with ATP, see below).

**Fig. 4:**
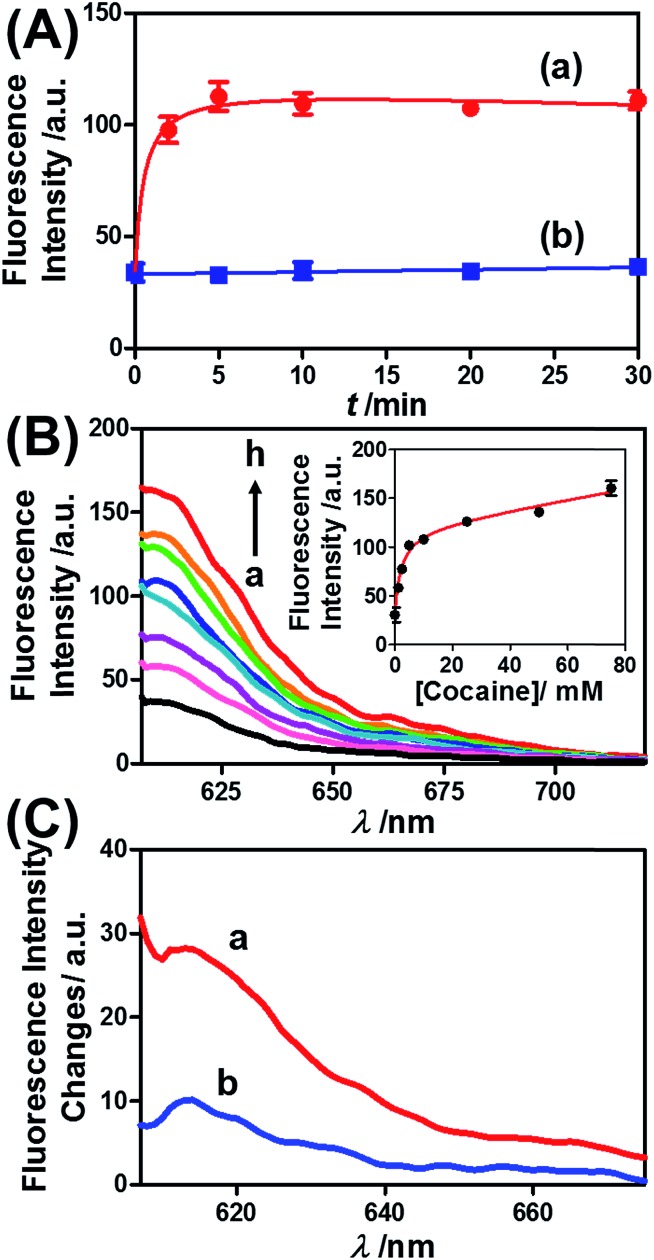
(A) Time-dependent release of TR-D from the cocaine-responsive hydrogel microcapsules treated with 10 mM cocaine (a) and without added cocaine (b). (B) Fluorescence spectra corresponding to the release of TR-D upon exposing the microcapsules loaded with TR-D to various concentrations of cocaine for a fixed time interval of 30 min: (a) 0, (b) 1.25, (c) 2.5, (d) 5, (e) 10, (f) 25, (g) 50 and (h) 75 mM. Inset: derived calibration curve of the fluorescence intensities of the released TR-D at *λ*_em_ = 610 nm upon treatment with different concentrations of cocaine. (C) Fluorescence spectra corresponding to the release of TR-D upon the treatment of the cocaine-responsive microcapsules with cocaine and its derivative for a fixed time interval of 30 min: (a) 2.5 mM cocaine and (b) 2.5 mM ecgonine methyl ester (crack).

Similar results were observed for the cocaine-responsive microcapsules. Fig. 4 depicts the cocaine-driven release of TR-D from the microcapsules crosslinked with (**5**)/(**3**)–(**6**) (for a microscopic characterization of the cocaine-responsive microcapsules, see Fig. S7†). Fig. 4(A) shows the time-dependent release of TR-D upon the treatment of the microcapsules with a fixed concentration of cocaine corresponding to 10 mM. As the release time was prolonged, the fluorescence intensity of the released load in the bulk solution increased, which indicated a greater extent of release. The fluorescence in the bulk solution leveled off after *ca.* 5 minutes, which indicated that after this time interval all the TR-D load was released. By knowing the concentration of the microcapsules from flow cytometry and using an appropriate calibration curve, we estimated that the average loading of TR-D in the cocaine-responsive microcapsule was 3.0 × 10^–14^ mol. Fig. 4(B) shows the fluorescence spectra of TR-D that was released upon the treatment of the loaded microcapsules with varying concentrations of cocaine for a fixed time interval of 30 minutes. As the concentration of cocaine increased, the intensity of the fluorescence spectra increased, which implied an enhancement of release. Fig. 4(B) (inset) depicts the resulting calibration curve. The cocaine-stimulated release of the TR-D load was selective, and the treatment of the cocaine-responsive microcapsules with the cocaine derivative ecgonine methyl ester or “crack” (which is not recognized by the aptamer) did not lead to the unlocking of the microcapsules and the release of TR-D (Fig. 4(C)). The results indicated that the selective formation of the cocaine–aptamer complex led to the dissociation of the (**5**)/(**6**) bridging units in a process that increased the permeability of the hydrogel shell, which allowed the release of TR-D from the microcapsules.

**Fig. 5:**
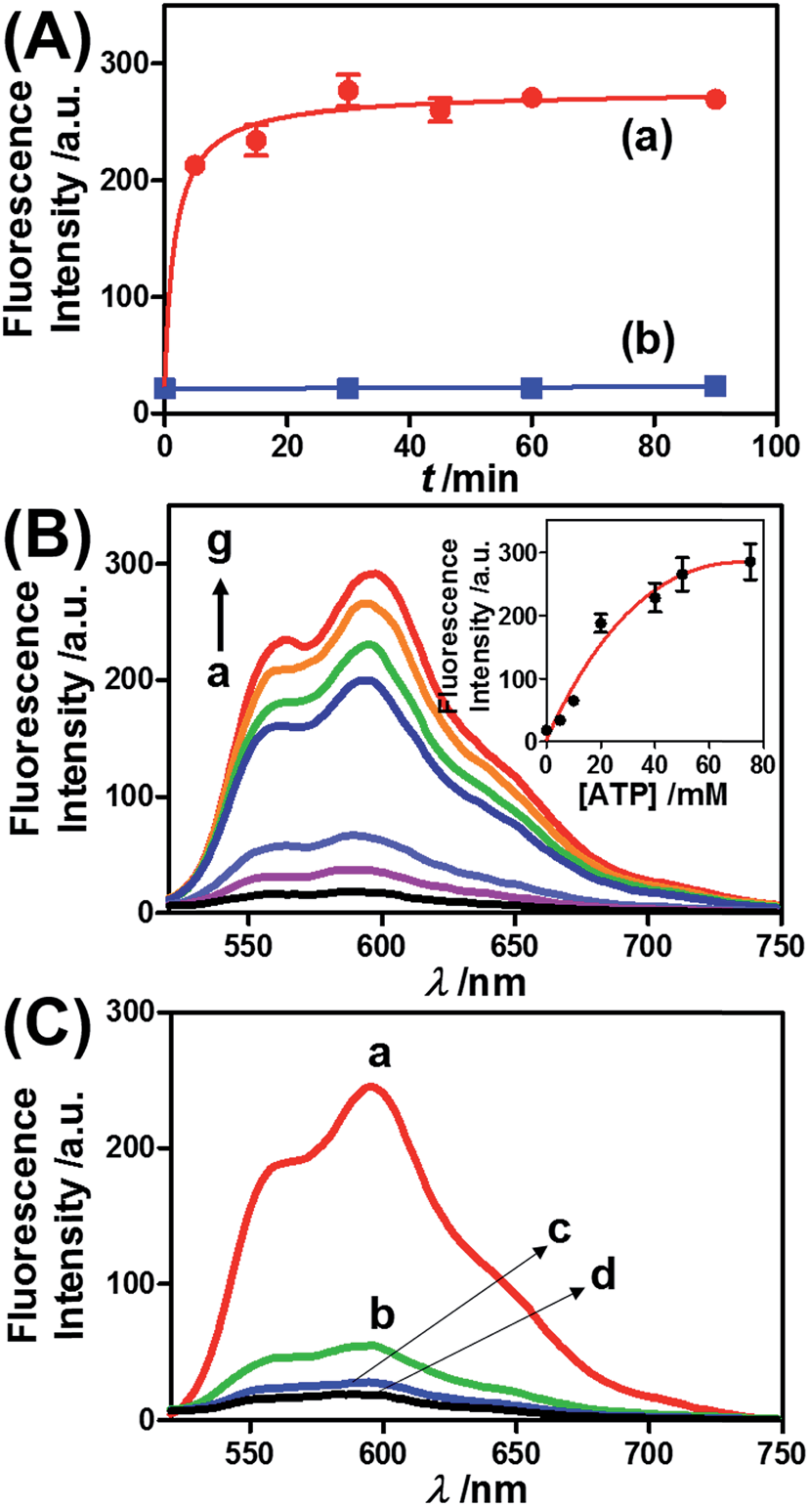
(A) Time-dependent release of DOX-D from the ATP-responsive hydrogel microcapsules treated with 50 mM ATP (a) and without added ATP (b). The released DOX-D was acidified to enable the cleavage of fluorescent doxorubicin from dextran. (B) Fluorescence spectra corresponding to the release of doxorubicin upon exposing the ATP-responsive hydrogel microcapsules to different concentrations of ATP for a fixed time interval of 30 min: (a) 0, (b) 5, (c) 10, (d) 20, (e) 40, (f) 50, and (g) 75 mM. Inset: derived calibration curve of the fluorescence intensities of the released doxorubicin at *λ*_em_ = 600 nm upon treatment with different concentrations of ATP. (C) Fluorescence spectra corresponding to the release of doxorubicin from the ATP-responsive microcapsules loaded with doxorubicin-dextran and exposed to different nucleoside triphosphates for a fixed time interval of 30 minutes: (a) 25 mM ATP, (b) 25 mM CTP, (c) 25 mM GTP, and (d) untreated.

ATP is overexpressed in cancer cells owing to the enhanced metabolism in these cells. Accordingly, we considered the possibility of loading the microcapsules with doxorubicin-modified dextran (DOX-D) (*cf.* Fig. 1), and examined the ATP-driven release of DOX-D as a chemotherapeutic prodrug. Note that doxorubicin was linked through a boronate ester group to the dextran backbone. The resulting DOX-D exhibited very low fluorescence intensity, but under the acidic conditions (pH ≈ 5.5–6.5) present in cancer cells, hydrolysis of the doxorubicin boronate ester proceeded to yield the highly fluorescent free doxorubicin (for a detailed characterization of DOX-D, see the experimental section and ESI†). Fig. 5 shows the ATP-driven release of DOX-D from the ATP-responsive microcapsules. In these experiments, the release of DOX-D from the microcapsules was examined at pH = 7.2, and the samples that contained the released DOX-D were acidified to hydrolyze the boronate ester to the fluorescent free doxorubicin, which enabled the spectroscopic analysis of the release of DOX-D from the microcapsules. Fig. 5(A) shows the time-dependent release of DOX-D from the microcapsules in the presence of a constant concentration of ATP (50 mM). The fluorescence intensity of the released DOX-D increased with time and reached a saturation value after *ca.* 30 minutes, which corresponded to the fluorescence of all the released DOX-D. By knowing the concentration of the microcapsules from flow cytometry, we estimated that the average loading of DOX-D in the microcapsules was 6.9 × 10^–17^ mol. Fig. 5(B) depicts the dependence of the release of DOX-D from the microcapsules as a function of the ATP concentration. In these experiments, the content of DOX-D released from the capsules was determined after a fixed time interval of 30 minutes in the presence of different concentrations of ATP. Fig. 5(B) (inset) shows the derived calibration curve. As the concentration of ATP increased, the release of DOX-D was enhanced, which was consistent with the increase in the permeability of the microcapsules as a result of unlocking the microcapsule shells *via* the formation of ATP–aptamer complexes. The unlocking of the microcapsules bridged by the ATP–aptamer using ATP was selective, and other nucleoside triphosphates did not dissociate the microcapsules and did not release DOX-D, as shown in Fig. 5(C).

**Fig. 6:**
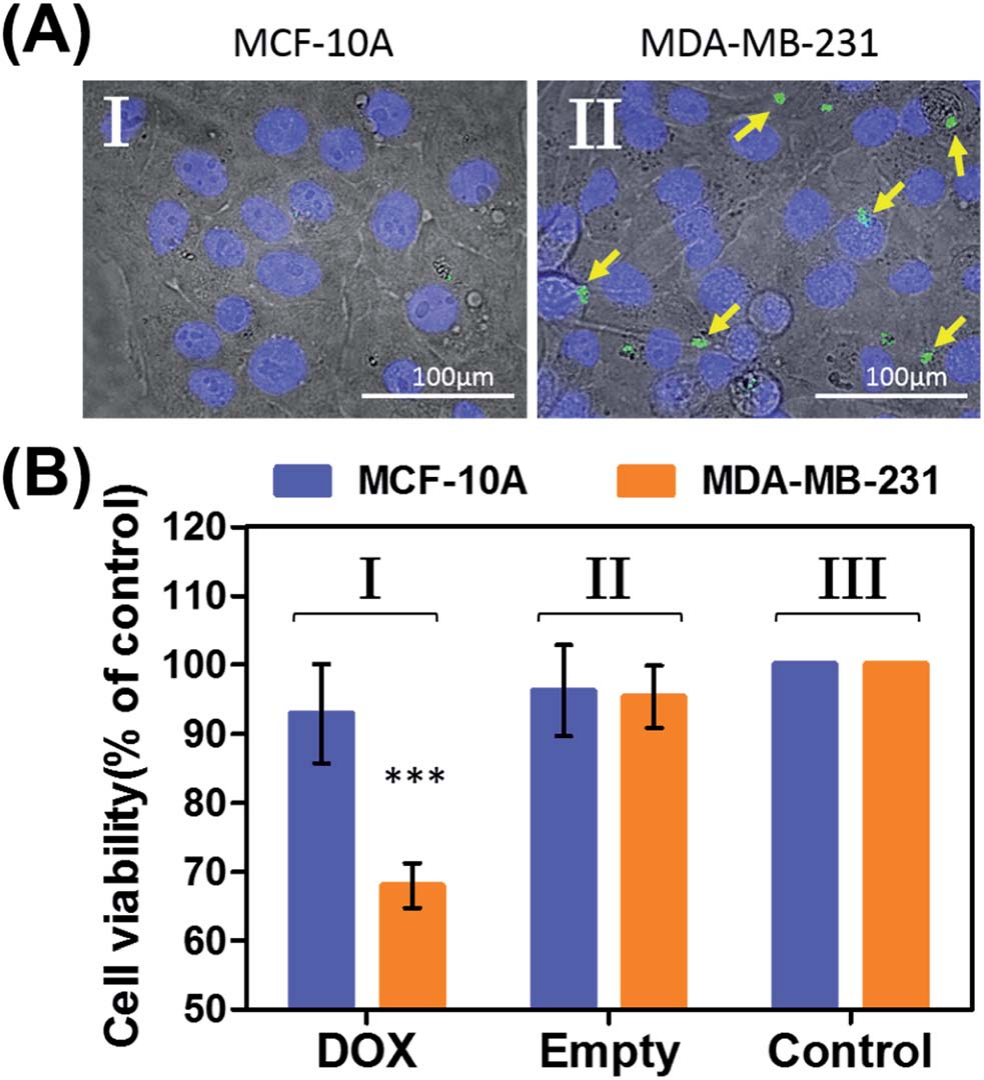
(A) Uptake of the ATP-responsive acrylamide hydrogel microcapsules loaded with CdSe/ZnS QDs emitting at 560 nm into MCF-10A normal breast epithelial cells and MDA-MB-231 malignant breast cancer cells. (I) Fluorescence and bright-field merged images of the uptake of the capsules into MCF-10A cells. (II) Fluorescence and bright-field merged images of the uptake of the capsules by malignant MDA-MB-231 cells. The nuclei were stained with Hoechst 33342 (appearing as blue). The yellow arrows indicate luminescent QDs in the cell compartments. (B) Cytotoxicity of the ATP-responsive microcapsules loaded with DOX-D toward MDA-MB-231 breast cancer cells and MCF-10A normal epithelial cells. The bars represent the cell viability after the treatment of the cells with the microcapsules for a time interval of 6 hours, followed by allowing cell growth for 48 hours. Entry I: the cells were treated with microcapsules loaded with DOX-D. Entry II: control experiments, where the cells were treated with drug-unloaded microcapsules. Entry III: control experiments, where the cells were not treated with microcapsules. *** denotes *p* < 0.001.

In the next step, we examined the possible unlocking of the ATP-responsive microcapsules in cancer cells and the release of DOX-D as an anti-cancer drug. In these experiments, we made use of the fact that ATP is overexpressed in cancer cells and that the release of this anti-cancer drug in cancer cells should be more efficient. Furthermore, the fact that cancer cells exhibit an acidic pH suggests that deprotection of DOX stabilized by a boronate ester should proceed in cancer cells, leading to the release of the free drug. Fig. 6 depicts the results of cytotoxicity studies that examined the effect of the ATP-responsive microcapsules loaded with DOX-D on MDA-MB-231 breast cancer cells and MCF-10A normal breast epithelial cells. Fig. 6(A) shows confocal microscopy images of MCF-10A breast epithelial cells (panel I) and MDA-MB-231 breast cancer cells (panel II) treated with the microcapsules loaded with CdSe/ZnS QDs emitting at 560 nm. Evidently, the permeation of the microcapsules loaded with QDs only proceeded into the MDA-MB-231 cancer cells. Although the mechanism of the selective permeation of the microcapsules is at present unknown, the enhanced permeation of the microcapsules (EPR effect) through the boundaries of cancer cells might explain the observed phenomenon. Fig. 6(B) depicts the effect of the ATP-responsive microcapsules loaded with DOX-D on MDA-MB-231 breast cancer cells, MCF-10A breast epithelial cells, and respective control systems. After a time interval of 48 hours, the viability of the MDA-MB-231 cells decreased by 35%, whereas the viability of normal cells decreased by only *ca.* 7%. The substantially enhanced cytotoxicity of the ATP-responsive microcapsules loaded with DOX-D to the breast cancer cells, in comparison to the normal cells, is attributed to the targeted permeation of the microcapsules loaded with DOX-D into the cancer cells.

**Fig. 7:**
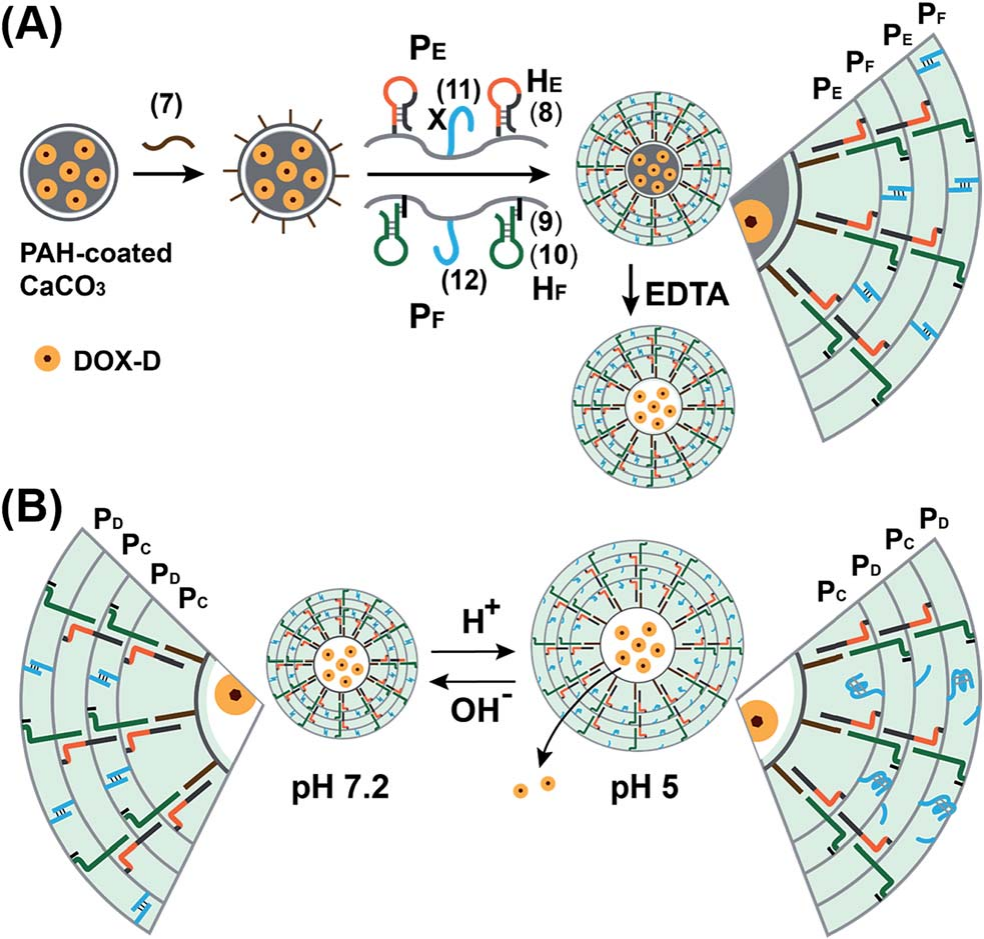
(A) Schematic of the pH-responsive hydrogel microcapsules using the promoter-induced hybridization chain reaction (HCR) process, which yielded an acrylamide hydrogel shell composed of the duplexes generated by the cross-opening of hairpins H_E_ and H_F_ and by the pH-responsive duplexes consisting of (**11**)/(**12**). (B) Schematic of the pH-driven release of a loaded drug from the hydrogel microcapsules bridged by the i-motif.

We further examined the design of pH-responsive copolymer–acrylamide hydrogel microcapsules, and investigated the possibility of implementing these systems as potential triggered carriers for chemotherapeutic drugs. Fig. 7(A) illustrates the method of preparing the pH-responsive hydrogel microcapsules, and a suggested mechanism of the pH-stimulated unlocking of the microcapsules and release of the encapsulated loads is shown in Fig. 7(B). Two acrylamide copolymer chains P_E_ and P_F_ were synthesized. The acrylamide copolymer chain P_E_ was functionalized with the hairpin H_E_ (**8**) and with the single-strand tether (**11**). The acrylamide copolymer chain P_F_ was modified with the duplex hairpin H_F_ (**9**)/(**10**) and with the nucleic acid tether (**12**). The tethers (**11**) and (**12**) exhibited base-pair complementarity, and the domain “X” in tether (**11**) was cytosine-rich and capable of forming the i-motif structure at pH = 5.0. Strand (**7**) was adsorbed onto the PAH-coated CaCO_3_ microparticles loaded with DOX-D, and this acted as an initiator strand to promote the hybridization chain reaction (HCR). In the presence of the polymer chains P_E_ and P_F_ and at pH = 7.2, the HCR process led to the coating of the microcapsules with the acrylamide copolymer hydrogel, which was cooperatively stabilized by the duplex formed by the cross-opening of hairpins H_E_ and H_F_ and by the duplexes formed between tethers (**11**) and (**12**). The dissolution of the CaCO_3_ microcapsule cores by EDTA yielded the hydrogel-stabilized microcapsules loaded with DOX-D. At an acidic pH, the duplex units (**11**)/(**12**) were separated owing to the formation of the i-motif structure within strand (**11**). The separation of the duplex (**11**)/(**12**) decreased the stiffness of the hydrogel shell, and the enhanced fluidity of the shell allowed the release of the DOX-D loads. For confocal microscopy/bright-field images and SEM images of the microcapsules see the ESI (Fig. S11 and S12†).

**Fig. 8:**
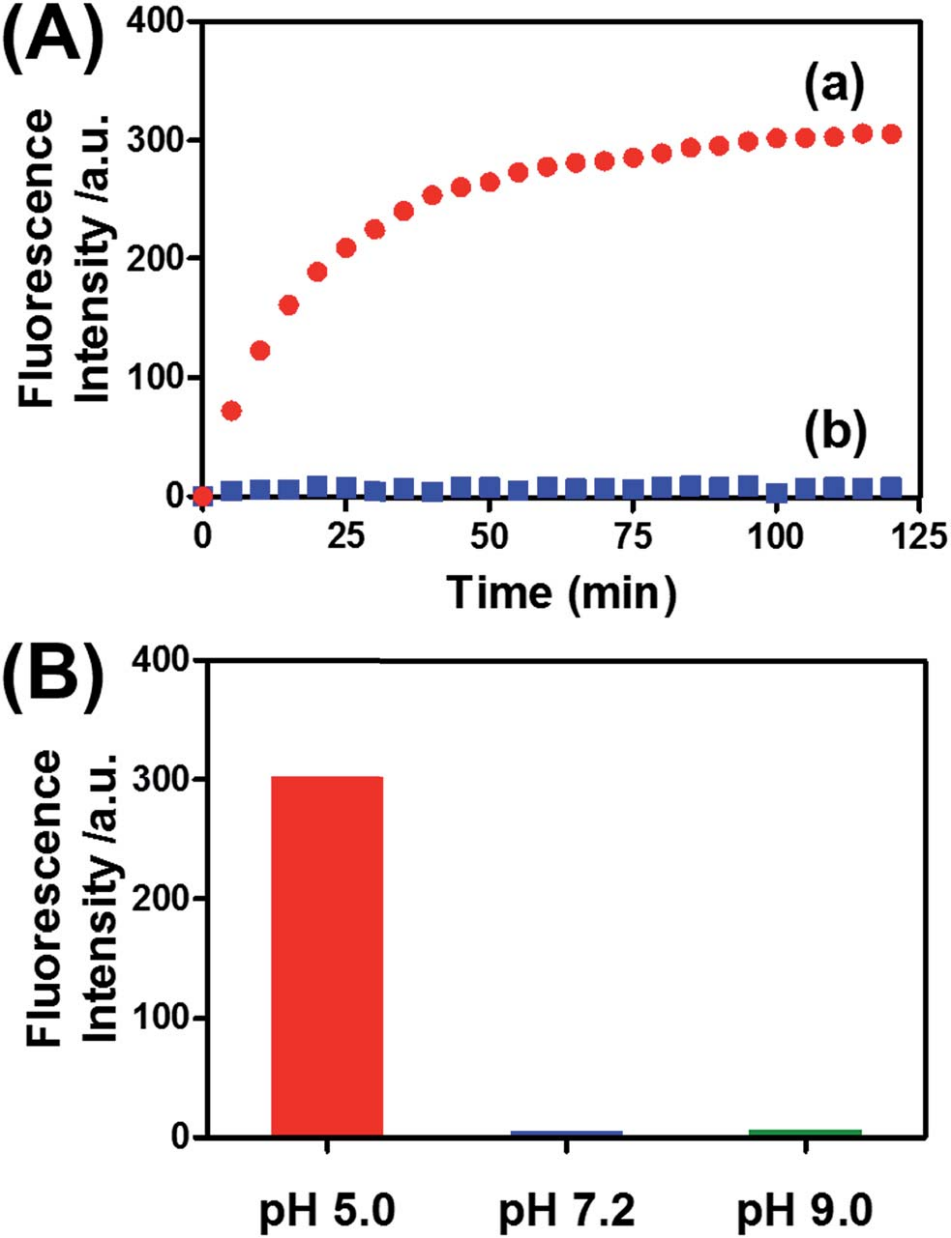
(A) Time-dependent release of doxorubicin from the pH-responsive hydrogel microcapsules. The microcapsules were subjected to a pH of 5.0 (curve a) and a pH of 7.2 (curve b). (B) Fluorescence intensities of the released doxorubicin corresponding to the microcapsules subjected to different pH values for a fixed time interval of 120 min.

Fig. 8(A) (curve (a)) depicts the time-dependent release of DOX-D from the microcapsules at pH = 5.0. The release of DOX-D increased with time, and the fluorescence of the released load leveled off after *ca.* 40 minutes. After this time interval, we assume that all the loaded DOX-D was released from the microcapsules. By knowing the concentration of the microcapsules and the total amount of released DOX, we find that the average loading of DOX-D in the microcapsules was 7.4 × 10^–17^ mol. Fig. 8(A) (curve (b)) shows the changes in fluorescence in the bulk solution upon subjecting the microcapsules loaded with DOX-D to pH = 7.2. No changes in fluorescence were observed, which implies that at pH = 7.2 the microcapsules were locked, and no DOX-D was released. These results imply that an acidic pH unlocks the microcapsules, presumably *via* the formation of the i-motif units, and thus allows the release of DOX-D. Fig. 8(B) depicts the release of DOX-D upon subjecting the microcapsules to different pH values. At a neutral or basic pH no release of DOX-D was observed, and acidification of the solution activated the release process and the release of the DOX-D load. Further support of the conclusion that acidification of the microcapsules led to the formation of the i-motif structure was obtained by circular dichroism experiments (Fig. S13†). At pH = 7.2, a bisignate CD spectrum of duplex DNA units bridging the hydrogel was observed. Treatment of the hydrogel at pH = 5.0 resulted in a red shift in the bisignate CD spectrum, which was consistent with the formation of i-motif structures in the hydrogel matrix.^24^

**Fig. 9:**
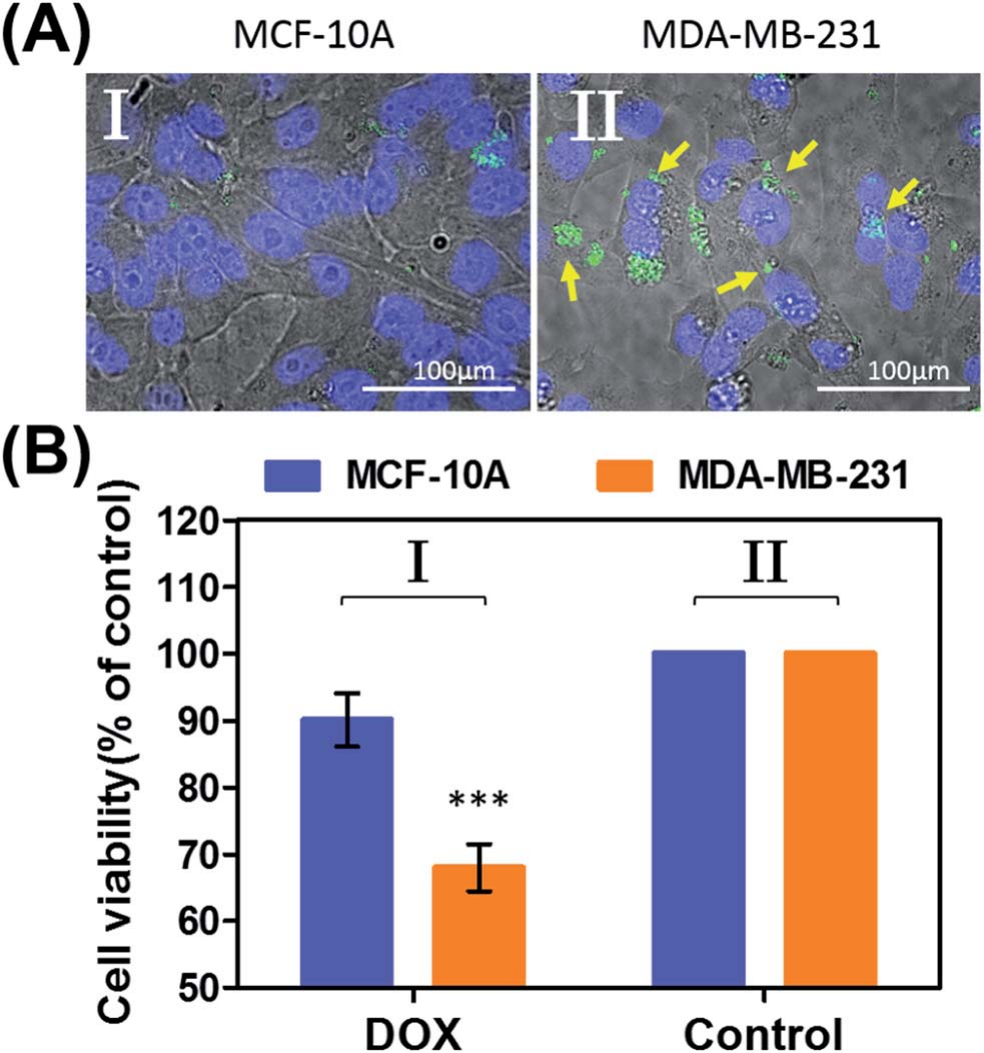
(A) Uptake of the pH-responsive acrylamide hydrogel microcapsules loaded with CdSe/ZnS QDs emitting at 560 nm into MCF-10A normal breast epithelial cells and MDA-MB-231 malignant breast cancer cells. (I) Fluorescence and bright-field merged images of the uptake of the capsules into MCF-10A cells. (II) Fluorescence and bright-field merged images of the uptake of the capsules by malignant MDA-MB-231 cells. The nuclei were stained with Hoechst 33342 (appearing as blue). The yellow arrows indicate luminescent QDs in the cell compartments. (B) Cytotoxicity of the pH-responsive microcapsules loaded with DOX-D toward MDA-MB-231 breast cancer cells and MCF-10A normal epithelial cells. The bars represent the cell viability after the treatment of the cells with the microcapsules for a time interval of 6 hours, followed by allowing cell growth for 5 days. Entry I: the cells were treated with microcapsules loaded with DOX-D. Entry II: control experiments, where the cells were not treated with microcapsules. *** denotes *p* < 0.001.

Preliminary studies examined the permeation features of the microcapsules loaded with QDs emitting at 560 nm into MCF-10A normal breast cells and MDA-MB-231 breast cancer cells (Fig. 9(A)). Although the permeation of the microcapsules loaded with QDs into the MCF-10A cells could not be detected (Fig. 9(A), panel I), the effective incorporation of the QDs into the cancer cells was detected (Fig. 9(A), panel II). In addition, the cytotoxicity of the pH-responsive microcapsules loaded with DOX-D to the normal and breast cancer cells was examined (Fig. 9(B)). After a time interval of five days, the MDA-MB-231 cancer cells treated with the microcapsules loaded with DOX-D exhibited a cell death rate of *ca.* 35%, whereas the normal MCF-10A cells displayed a cell death rate of only *ca.* 8%. These results are consistent with the enhanced permeation of the microcapsules into the cancer cells.

**Fig. 10:**
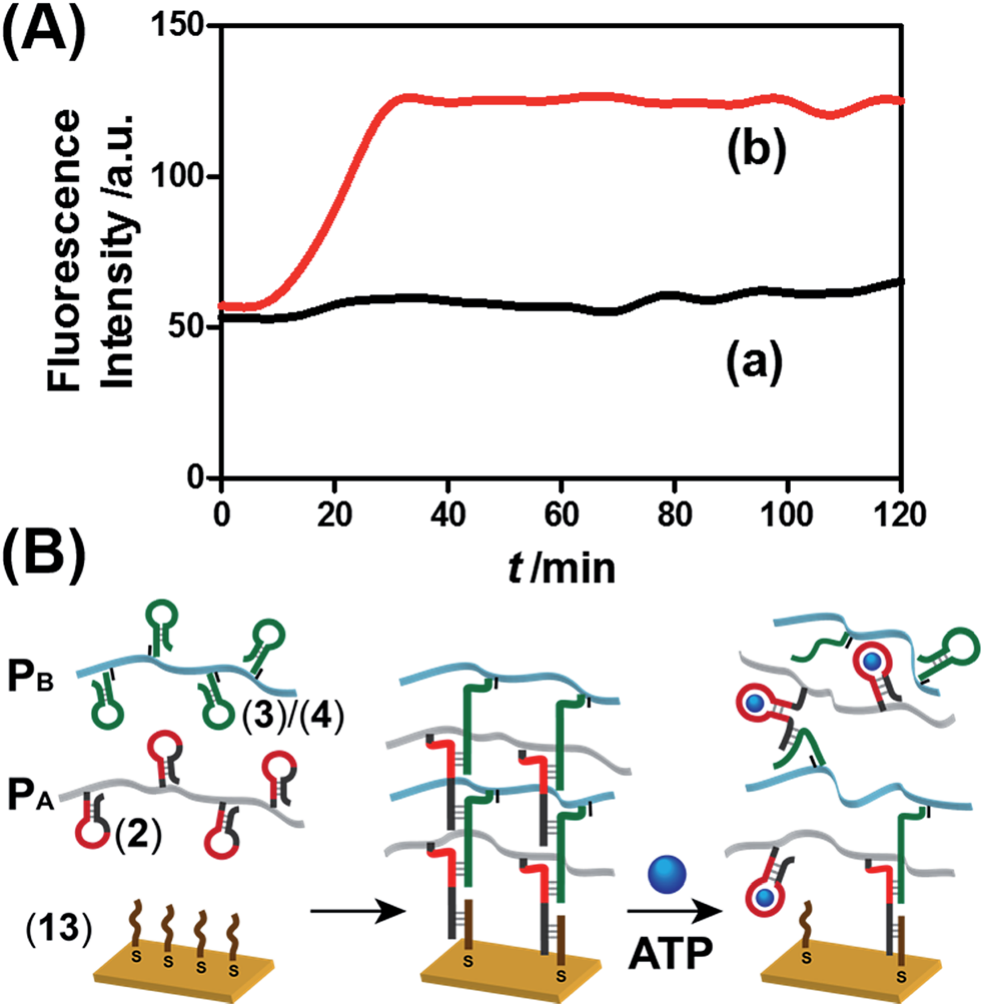
(A) Time-dependent release of TMR-D from the ATP-responsive hydrogel matrix in the absence of ATP (curve a) and in the presence of 50 mM ATP (curve b). (B) Schematic of the preparation of the hydrogel membranes on a gold-coated surface and the separation of the hydrogel matrix by the ATP trigger.

The ATP- and pH-driven release of the loads from the acrylamide hydrogel microcapsules crosslinked by an aptamer or i-motif was further confirmed by characterizing the permeability of the loads through the stimuli-responsive membranes and by determining the diffusion properties (Young's moduli) of the hydrogel membranes before and after the application of the release-triggering signals. Fig. 10(A) depicts the time-dependent release of TMR-D from the bulk hydrogel matrix crosslinked by (**2**)/(**4**) in the absence of the ATP trigger (curve (a)) and upon the treatment of the hydrogel with ATP (50 mM) (curve (b)). No release of the load was observed in the absence of ATP, which implied that the rigid structure of the hydrogel inhibited the permeation of the load through the hydrogel. In turn, treatment of the hydrogel with ATP resulted in the release of the dye (Fig. 10(A), curve (b)), which demonstrated that the formation of the ATP–aptamer complex dissociated the crosslinking nucleic acid bridges in part, which resulted in a reduction in the stiffness of the polymer matrix that allowed the release of the load. In addition, a thin film (thickness of *ca.* 50 μm) of the acrylamide hydrogel crosslinked by (**2**)/(**4**) was assembled on a gold-coated surface following a recently developed method reported by our laboratory.^22^ A primer nucleic acid (**13**) was immobilized on a gold-coated surface. The promoter strand (**13**) initiated, in the presence of the acrylamide copolymer chains P_A_ and P_B_ modified with the hairpins H_A_ and H_B_, the hybridization chain reaction, which yielded a crosslinked hydrogel film that included in the duplex units the anti-ATP aptamer sequences in caged configurations (Fig. 10(B)). The resulting film exhibited a Young's modulus of 94 ± 24 Pa. After treatment of the film with ATP (100 mM), the value of the Young's modulus decreased to 20 ± 11 Pa, which was consistent with the separation of the nucleic acid bridging unit *via* the formation of the ATP/aptamer complex and the formation of a hydrogel of low stiffness (or higher fluidity). Similar results were observed upon treatment of the cocaine-responsive bulk hydrogel or cocaine-responsive hydrogel film on a surface using cocaine as a trigger (Fig. S14, ESI†).

Similar results were observed for the pH-responsive hydrogel matrix (Fig. S15, ESI†). TMR-D dye was incorporated in a piece of hydrogel composed of the acrylamide copolymer chains P_E_ and P_F_ crosslinked by H_E_, H_F_ and the pH-responsive bridging units (**11**)/(**12**). The hydrogel matrix crosslinked by (**8**)/(**10**) and (**11**)/(**12**) did not allow the release of the dye from the hydrogel matrix (Fig. S15,† curve (a)), which demonstrated the stiffness of the polymer matrix. In turn, acidification of the hydrogel at pH = 5.0 resulted in the release of the dye load from the hydrogel matrix (Fig. S15,† curve (b)). These results indicate that the acidification of the hydrogel dissociated the pH-responsive crosslinking units, which led to a reduction in the stiffness of the hydrogel that allowed the release of the load. Similarly, a thin film of the acrylamide copolymer hydrogel crosslinked by (**8**)/(**10**) and (**11**)/(**12**) was deposited on a gold-coated surface (Fig. S15(B)†). The Young's modulus of the hydrogel crosslinked by (**8**)/(**10**) and (**11**)/(**12**) corresponded to 83 ± 10 Pa. In turn, acidification of the system and separation of the i-motif bridging units resulted in a reduction in the stiffness of the hydrogel and an increase in fluidity, with a Young's modulus of 19 ± 6 Pa.

## Conclusions

The present study has introduced a versatile method of preparing stimuli-responsive polyacrylamide hydrogel microcapsules crosslinked by nucleic acids. In contrast to previous studies that demonstrated the preparation of all-DNA stimuli-responsive microcapsules, the present study introduced polyacrylamide as the major ingredient of the microcapsule shells and stimuli-responsive nucleic acids only as minor crosslinking elements of the hydrogel shells. Besides their cost-effectiveness, the resulting microcapsules displayed enhanced stability against enzymatic degradation, in comparison to all-DNA microcapsules, owing to the shielding of the nucleic acid elements by the polyacrylamide shell. The stimuli-responsive nucleic acid crosslinking units, which stabilize the hydrogel shells of the microcapsules, allow the triggered release of loads encapsulated in the microcapsules. The generic method of preparing the microcapsules involved the functionalization of substrate-loaded CaCO_3_ microcapsules with nucleic acid promoter units, which activated the hybridization chain reaction in the presence of two polyacrylamide chains P_i_ and P_j_ functionalized with two nucleic acid hairpin structures H_i_ and H_j_ (and optionally additional stimuli-responsive acid tethers) to yield hydrogel coatings on the loaded CaCO_3_ microparticle templates. The subsequent dissolution of the CaCO_3_ core yielded the substrate-loaded stimuli-responsive microcapsules. In the presence of appropriate triggers, the permeability across the microcapsule shells was facilitated by decreasing the stiffness of the shell membrane, which resulted in the triggered release of the loads. Specifically, we demonstrated the triggered release of loads induced by ATP or cocaine *via* the separation of the nucleic acid duplex bridging units crosslinking the polyacrylamide hydrogel chains by the formation of ATP–aptamer or cocaine–aptamer complexes. Similarly, pH-responsive hydrogel microcapsules were synthesized, and the triggered pH-stimulated release of the loads was demonstrated by the pH-induced separation of the nucleic acid duplex units bridging the polyacrylamide chains *via* the formation of an i-motif structure (pH = 5.0). Preliminary experiments revealed the selective cytotoxicity of ATP-responsive or pH-responsive microcapsules loaded with doxorubicin-dextran to MDA-MB-231 breast cancer cells.

Many other switchable stimuli-responsive DNA structures are known, *e.g.*, K^+^ ion/crown ether G-quadruplexes and the pH-induced formation of DNA triplexes, as well as the light-induced separation of nucleic acid duplexes in the presence of photoisomerizable compounds. In addition, the stiffness of hydrogel membranes may be controlled by different DNA architectures such as i-motif or G-quadruplex “springs”. Furthermore, other backbone polymers besides polyacrylamide chains can be used to yield the stimuli-responsive microcapsules crosslinked by DNA. All these strategies provide a rich arsenal of elements that could be employed to tailor substrate-loaded stimuli-responsive hydrogel microcapsules. Such microcapsules are anticipated to act as carriers for the controlled release of drugs or the ingredients of cosmetics and fragrances.

## Experimental section

### Reagents and materials

Magnesium chloride, sodium chloride, doxorubicin hydrochloride (DOX), 4-carboxyphenylboronic acid, dextran (MW = 40 kDa), 4-(2-hydroxyethyl)piperazine-1-ethanesulfonic acid sodium salt (HEPES base), 4-(2-hydroxyethyl)piperazine-1-ethanesulfonic acid (HEPES acid), *N*-(3-dimethylaminopropyl)-*N*′-ethylcarbodiimide hydrochloride (EDC), *N*-hydroxysulfosuccinimide sodium salt (sulfo-NHS), poly(acrylic acid) (MW = 30, 450, and 1250 kDa), 2-(*N*-morpholino)ethanesulfonic acid (MES), ammonium persulfate (APS), *N*,*N*,*N*′,*N*′-tetramethylethylenediamine (TEMED), acrylamide solution (40%), poly(allylamine hydrochloride) (PAH, MW = 58 kDa), ethylenediaminetetraacetic acid disodium salt dihydrate (EDTA), adenosine triphosphate (ATP), guanosine triphosphate (GTP), cytidine triphosphate (CTP), and thymidine triphosphate (TTP) were purchased from Sigma-Aldrich. Tetramethylrhodamine-dextran (TMR-D, MW = 70 kDa) and Texas Red-dextran (TR-D, MW = 70 kDa) were purchased from Life Technologies Corporation (USA). All oligonucleotides were synthesized, purified by standard desalting, and freeze-dried by Integrated DNA Technologies, Inc. (Table S1, ESI†). Ultrapure water from a NANOpure Diamond (Barnstead International, Dubuque, IA) source was used in all experiments. A Magellan XHR 400L scanning electron microscope (SEM) and an FV-1000 confocal microscope (Olympus, Japan) were employed to characterize the microparticles.

### Synthesis of doxorubicin-conjugated dextran

Doxorubicin-conjugated dextran was synthesized by the modification of doxorubicin with *p*-aminophenylboronic acid and linking the conjugate to dextran *via* boronate ester bonds (ESI, Fig. S10†). 4-Carboxyphenylboronic acid solution (1.4 mL, 0.5 mM in 10 mM HEPES, pH 7.0, including 500 mM NaCl) was mixed with EDC (0.8 mg, 4.1 μmol) and sulfo-NHS (2.2 mg, 10.1 μmol) and allowed to react at room temperature for 15 min. Subsequently, doxorubicin (100 μL, 10 mM) was added to the mixture to react at room temperature for 2 h with continuous shaking, followed by incubation at 4 °C overnight. Dextran solution (2 mL, 0.08 mg mL^–1^ in 10 mM HEPES, pH 10.0) was added to the doxorubicin solution functionalized with boronic acid and reacted at room temperature for 2 h with continuous shaking, followed by incubation overnight at 4 °C. Free doxorubicin was removed using a centrifugal filter device (Amicon, 10k MWCO). The purified doxorubicin-dextran conjugate was stored at 4 °C until use. Then, a 40 μM doxorubicin-conjugated dextran solution was prepared by dissolving 0.32 mg DOX-D in 100 μL buffer solution. The average loading of doxorubicin on a dextran chain was 60 (60 doxorubicin units per chain), which was determined by measuring the fluorescence intensity of the DOX released by hydrolysis at a pH of 5. The released amount of DOX was deduced from a calibration curve of the fluorescence of known concentrations of DOX.

### Synthesis of poly(acrylic acid) modified with the promoter oligonucleotide

EDC (2 mg) and sulfo-NHS (5.5 mg) were added to 100 μL of a 10 mg mL^–1^ solution of poly(acrylic acid) (30 kDa) (prepared in 0.1 M MES, pH 5.5), and the mixture was allowed to react for 15 min at room temperature. Then, 1 mL of a 100 μM solution of the promoter oligonucleotide (**1a** or **1b**) in 0.1 M phosphate buffer (pH 7.2) was added to the mixture and reacted at room temperature for 2 h, followed by incubation at 4 °C overnight. The polymers modified with the promoter oligonucleotide, namely, PAA-(**1a**) and PAA-(**1b**), were purified through an Amicon filter (10 kDa MWCO) to remove unconjugated oligonucleotides.

### Synthesis of acrydite-modified oligo/acrylamide copolymers

Firstly, 100 μL of a solution consisting of 0.75 mM acrydite-modified oligonucleotide (**2**, **3**, or **5**) and 1.5% acrylamide was bubbled with nitrogen gas for 3 min, and then 7.5 μL of an initiator mixture (prepared as 10 mg APS in 5 μL TEMED and 95 μL H_2_O) was added to the solution. For the pH-responsive system, 100 μL of a solution consisting of an acrydite-modified oligonucleotide (0.75 mM (**8**)/1.5 mM (**11**) or 0.75 mM (**9**)/1.5 mM (**12**)) and 1.5% acrylamide was bubbled with nitrogen gas for 3 min, and then 7.5 μL of an initiator mixture was added. The solutions were subjected to an additional 10 min of nitrogen bubbling, followed by incubation at 4 °C for 12 h to form the copolymer chains. Polymers P_A_ (**2**) and P_C_ (**5**) were purified and separated from the unreacted compounds by a centrifugal filter device (Amicon, 30k MWCO), whereas polymers P_B_ (**3**), P_E_ (**8** and **11**) and P_F_ (**9** and **12**) (before hybridization of (**10**)) were filtered by a 10k MWCO Amicon filter. After being washed with water five times, the copolymer solutions were dried under a gentle flow of nitrogen gas and redispersed in a buffer (25 mM HEPES, pH 7.2, containing 25 mM MgCl_2_ and 10 mM NaCl). Hybridization of hairpins (**4**) and (**6**) with the polymer-incorporated (**3**) to form P_B_ and P_D_ and hybridization of hairpin (**10**) with the polymer-incorporated (**9**) to form P_F_ were performed after the formation of the polymer. After the determination of the concentration of (**3**) and (**9**), hairpins (**4**), (**6**) and (**10**) were added in a molar ratio of 1 : 1. The polymer solutions were incubated at 95 °C for 5 min, followed immediately by incubation on ice for 30 min to ensure the efficient closing of the hairpins.

### Preparation of CaCO_3_ microparticles with different loads

The preparation of CaCO_3_ microparticles followed a previously reported method.^17^ The loads of TMR-D, TR-D and DOX-D were 0.2, 0.2, and 0.3 mg mL^–1^, respectively. The resulting loaded CaCO_3_ microparticles were used as templates to assemble the microcapsules.

### Synthesis of DNA–acrylamide hydrogel microcapsules

CaCO_3_ microparticles (6.0 mg) were suspended in 300 μL of a 1 mg mL^–1^ solution of PAH (10 mM HEPES, pH 7.2, containing 500 mM NaCl and 50 mM MgCl_2_) and kept under continuous shaking for an adsorption time interval of 30 min. The PAH-coated particles were washed twice with a buffer (25 mM HEPES, pH 7.2, containing 25 mM MgCl_2_ and 10 mM NaCl), followed by centrifugation at 900 rpm for 20 s. Subsequently, the PAH-coated microparticles were incubated with 300 μL of PAA conjugated with the promoter nucleic acids (**1a** or **1b**) (the final concentration of the promoter nucleic acids was 10 μM) or 10 μM promoter nucleic acid (**7**) and kept under continuous shaking for an adsorption time interval of 30 min. After being washed twice with a buffer (25 mM HEPES, pH 7.2, containing 25 mM MgCl_2_ and 10 mM NaCl), followed by centrifugation at 900 rpm for 20 s, the DNA hydrogel particles were prepared by mixing each of the polymer sets (P_A_/P_B_, P_C_/P_D_ or P_E_/P_F_) immediately before adding the solution to the CaCO_3_ particles coated with the promoter. The final concentration of each hairpin was 10 μM. The particles were incubated at room temperature under continuous shaking for 12 hours, followed by centrifugation at 900 rpm for 20 s to remove non-adsorbed polymers. The DNA–acrylamide hydrogel particles that were formed were suspended in a buffer solution (480 μL, 25 mM HEPES, pH 7.2, containing 25 mM MgCl_2_ and 10 mM NaCl). Then, 120 μL of a 0.5 M solution of EDTA (pH 7.5) was introduced into the solution of the particles and incubated for 1 h to dissolve the CaCO_3_ cores. When the suspension became clear, the resulting capsules were washed with a buffer (25 mM HEPES, pH 7.2, containing 25 mM MgCl_2_ and 10 mM NaCl) using slow centrifugation (500 rpm, 20 min) three times.

### Stimuli-induced unlocking of the hydrogel microcapsules and release of the encapsulated loads

The concentrations of the solutions of the capsules employed in the experiments were 500 capsules per μL for the ATP-responsive microcapsules loaded with TMR-D and the cocaine-responsive microcapsules loaded with TR-D and 1500 capsules per μL for the ATP-responsive microcapsules loaded with DOX-D and the pH-responsive microcapsules loaded with DOX-D. Samples of 2 μL ATP or cocaine of various concentrations were added to 38 μL samples of the solutions of ATP-responsive or cocaine-responsive microcapsules. Then, 40 μL pH-responsive microcapsules were incubated with buffers of different pH values. After the desired incubation time intervals, the solutions of the capsules were centrifuged at 500 rpm (≈30 rcf) for 20 min to precipitate the residual capsules. Hydrolysis of DOX-D was performed by adding 5 μL hydrochloric acid to 30 μL of the DOX-D supernatant before measuring the fluorescence of doxorubicin. The fluorescence of the released TMR-D, TR-D, and doxorubicin was measured by a Cary Eclipse fluorescence spectrophotometer (Varian, Inc.).

### Cell culture

Normal breast epithelial cells (MCF-10A) used as controls were grown at 37 °C in a complete growth medium consisting of a 1 : 1 mixture of Dulbecco's modified Eagle's medium and Ham's F12 medium supplemented with 5% horse serum, 20 ng mL^–1^ epidermal growth factor, 0.1 μg mg^–1^ cholera toxin (CT), 10 μg mL^–1^ insulin, 500 ng mL^–1^ hydrocortisone and 1 unit per mL penicillin/streptomycin. Malignant breast epithelial cells (MDA-MB-231) were grown at 37 °C under 5% CO_2_ in RPMI 1640 medium supplemented with 10% fetal calf serum, l-glutamine, and antibiotics containing penicillin, streptomycin, and amphotericin B (Biological Industries). Both cell types were plated 1 day before experimentation onto 24-well plates or microscope slides attached to perforated tissue culture plates with a diameter of 3 cm for cell viability or microscopy measurements.

### Uptake of micro-capsules into cells

Normal breast cells (MCF-10A) and malignant breast epithelial cells (MDA-MB-231) were plated in a glass-bottomed Petri dish and incubated with 7.5 × 10^5^ micro-capsules per mL CdSe/ZnS QDs and doxorubicin-loaded micro-capsules in a growth medium for 6 hours. Then, the cells were intensively washed with DMEM-HEPES and incubated with Hoechst 33342 to stain their nuclei. The fluorescence of the CdSe/ZnS QDs was measured by epi-fluorescence microscopy (Nikon TE2000 microscope) equipped with an Opti-Grid. Image analysis was performed using the ImageJ and Volocity programs.

### Cell viability

Cell viability was assayed after the incubation of doxorubicin-loaded capsules with MCF-10A and MDA-MB-231 cells, which were plated at a density of 1.8 × 10^5^ cells per well in 24-well plates. After culturing the cells overnight, the cells were incubated with or without doxorubicin-loaded micro-capsules for 6 hours. Following intensive washing, the cells were further incubated for 2 or 5 days with a growth medium, and then the cell viability was determined using the fluorescent redox probe Alamar Blue. The fluorescence of Alamar Blue was recorded with a plate reader (Tecan Safire) after incubation for 1 hour at 37 °C (*λ*_ex_ = 560 nm; *λ*_em_ = 590 nm).

### Preparation of bulk hydrogel matrix

A 100 μL sample of a mixture consisting of the copolymer sets (P_A_/P_B_, P_C_/P_D_ or P_E_/P_F_) and 0.5 mg mL^–1^ TMR-D or TR-D was incubated with 25 μM promoter nucleic acid (**1a**, **1b**, or **7**) at room temperature overnight to form the hydrogel matrix. The final concentration of each hairpin was 100 μM.

### Stimuli-induced dissolution of the hydrogel matrix and release of the fluorescent loads

The resulting hydrogel matrix was washed with a buffer to remove unloaded fluorescent dextran. Then, 100 μL hydrogel matrix was introduced into a quartz cuvette and 400 μL buffer was added. ATP or cocaine was added to the solution, and the final concentrations of ATP and cocaine were 50 mM and 20 mM, respectively. For the pH-responsive hydrogel matrix, 400 μL HEPES buffer (pH 5.0 or 7.2) was added to 100 μL hydrogel matrix. The time-dependent dissolution of the hydrogel matrix and the fluorescence intensity of the released fluorescent dextran were measured by a Cary Eclipse fluorescence spectrophotometer (Varian, Inc.).
