# Supplementary material for: pH- and ligand-induced release of loads from DNA–acrylamide hydrogel microcapsules
Source: Chem Sci. 2017 Jan 10;8(5):3362–73. doi: 10.1039/c6sc04770j (PMC5416914; doi:10.1039/c6sc04770j)
Supplement: Supplementary file 1 [file SC-008-C6SC04770J-s001.pdf]

Electronic Supplementary Material (ESI) for Chemical Science.
This journal is © The Royal Society of Chemistry 2017

Supporting Information

pH- and Ligand-Induced Release of Loads from DNA-Acrylamide Hydrogel Microcapsules

Wei-Ching Liao,[a,‡] *Sivan Lilienthal,*[a,‡] *Jason S. Kahn,*[a] *Marianna Riutin,* [a] *Yang Sung Sohn,* [b] *Rachel Nechushtai,*[b] *and Itamar Willner* [a,*]

[a]Institute of Chemistry, Center for Nanoscience and Nanotechnology, The Hebrew University of Jerusalem, Jerusalem 91904, Israel

[b]Institute of Life Science, The Hebrew University of Jerusalem, Jerusalem 91904, Israel

‡These authors contributed equally to this work.

Table S1. The nucleic acid sequences used in this study.

No.	Sequence (5’→3’)
1a	/5AmMC6/TTTTTTAGCTGACCTGGGGGAG
1b	/5AmMC6/TTTTTTAGCGTAGGGAGACAAG
2	/5Acryd/TTTTTTTTCCTGGGGGAGTATTGCGGAGGAAGGGGATGTC TCCCCCAGGTCAGCT
3	/5Acryd/TGCTCTAGATCTGGTA
4	ACATCCCCTTCCTCCGAGCTGACCTGGGGGAGTATTGCGGAGGA AGGTACCAGATCTAGAGC
5	/5Acryd/TTTTTTTTGGGAGACAAGGAAATCCTTCAATGAAGTGGGT CGACACTTGTCTCCCTACGCT
6	TGTCGACCCACTTCATAGCGTAGGGAGACAAGTATTGATGAAGT GGGTACCAGATCTAGAGC
7	GTAGAAGAAGGTGTCACAGTT
8	/5Acryd/TTTTTTTTTTGGTGTTTAAGTTGGAGAATTGTACTTAAACA CCTTCTTCT
9	/5Acryd/TTTGGACCGATGTT AGA GC
10	CAATTCTCCAACTTAAACTAGAAGAAGGTGTTTAAGTTGGGCTCT AACATCGGTCCAA
11	/5Acryd/TTTCCCAATCCCAATCCCAATCCC
12	/5Acryd/TTTGTGTTAGGTTTAGGGTTAGTG
13	/5ThioMC6-D/TTTTTTAGCTGACCTGGGGGAG
14	/5ThioMC6-D/TTTTTTAGCGTAGGGAGACAAG
15	/5ThioMC6-D/TTTTTGTAGAAGAAGGTGTCACAGTT

Determination of Molecular Weight and Ratio of Acrylamide/Acrydite-Nucleic Acids

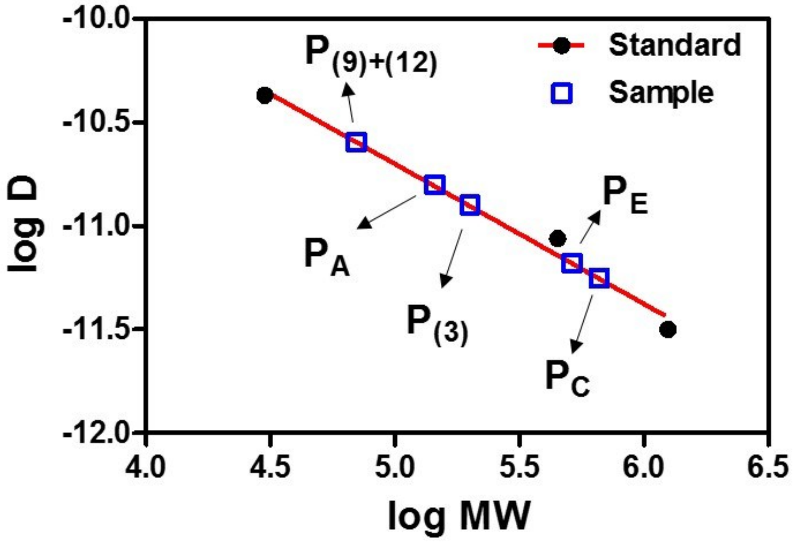

Figure S1. Calibration curve of the diffusion coefficients against known molecular weights of poly(acrylic acid) (30,000, 450,000, and 1,250,000 Da). The linear equation is y=-0.6751x-7.325 (R^2 = 0.9835). The diffusion coefficient is derived from the diffusion-ordered spectroscopy (DOSY) spectrum. Circles represent the known polymers utilized to form standard line, and squares represent the polymers measured for experimental studies.

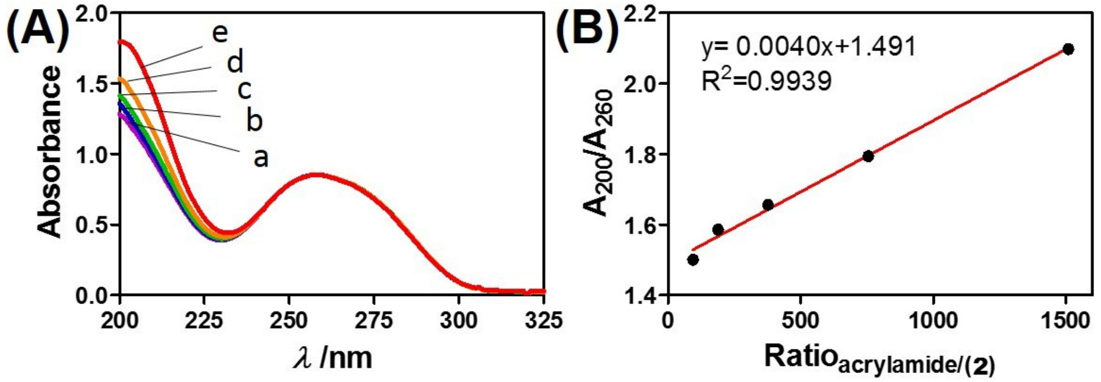

Figure S2. Determination of the ratio between nucleic acid units and acrylamide monomer units on the hairpin (**2**) conjugated copolymer P_A. (A) Absorbance spectra corresponding to various concentrations of polyacrylamide (5000 kDa, *ca.* 70343 monomers) in the presence of constant concentration of the acrydite-modified nucleic acid (**2**), 1.6 μM: (a) 2.3, (b) 4.5, (c) 8.9, (d) 17.8, and (e) 35.6 nM. (B) Calibration curve between the molar ratio of acrylamide monomer/(**2**) and the ratio of absorbance

at wavelength of 200 nm and 260 nm.

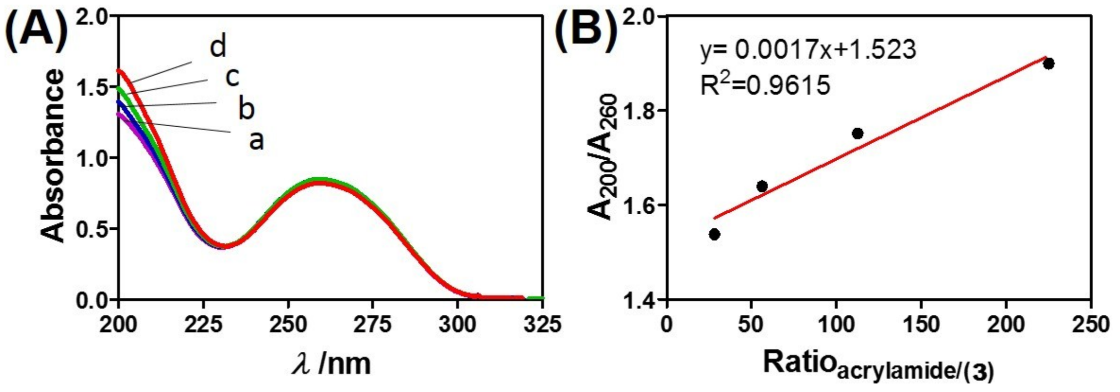

Figure S3. Determination of the ratio between nucleic acid units and acrylamide monomer units on the nucleic acid (**3**) conjugated copolymer P_B or P_D. (A) Absorbance spectra corresponding to various concentrations of polyacrylamide (5000 kDa, *ca.* 70343 monomers) in the presence of constant concentration of the acrydite-modified nucleic acid (**3**), 5.6 μM: (a) 2.3, (b) 4.5, (c) 8.9, and (d) 17.8 nM. (B) Calibration curve between the molar ratio of acrylamide monomer/(**3**) and the ratio of absorbance at wavelength of 200 nm and 260 nm.

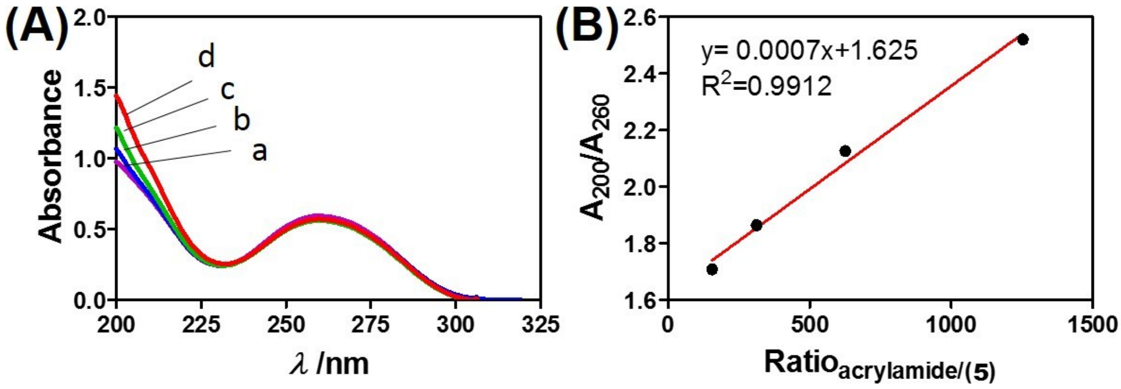

Figure S4. Determination of the ratio between nucleic acid units and acrylamide monomer units on the hairpin (**5**) conjugated copolymer P_C. (A) Absorbance spectra corresponding to various concentrations of polyacrylamide (5000 kDa, *ca.* 70343 monomers) in the presence of constant concentration of the acrydite-modified nucleic acid (**5**), 1.0 μM: (a) 2.3, (b) 4.5, (c) 8.9, and (d) 17.8 nM. (B) Calibration curve between the molar ratio of acrylamide monomer/(**5**) and the ratio of absorbance at wavelength of 200 nm and 260 nm.

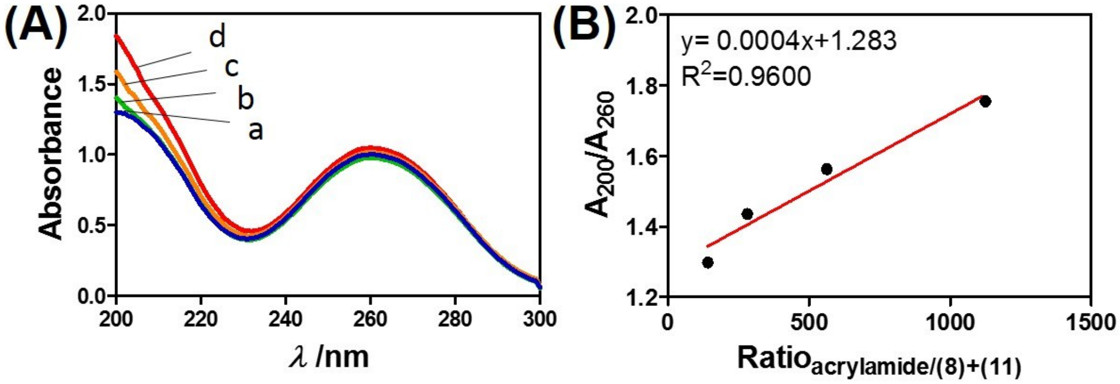

Figure S5. Determination of the ratio between nucleic acid units and acrylamide monomer units on the hairpin (**8**) and tether (**11**) conjugated copolymer P_E. (A) Absorbance spectra corresponding to various concentrations of polyacrylamide (5000 kDa, *ca.* 70343 monomers) in the presence of constant concentration of the acrydite-modified nucleic acid (**8**), 0.75 μM and (**11**), 1.5 μM: (a) 4.5, (b) 8.9, (c) 17.9, and (d) 35.7 nM. (B) Calibration curve between the molar ratio of acrylamide monomer/(**8**)+(**11**) and the ratio of absorbance at wavelength of 200 nm and 260 nm.

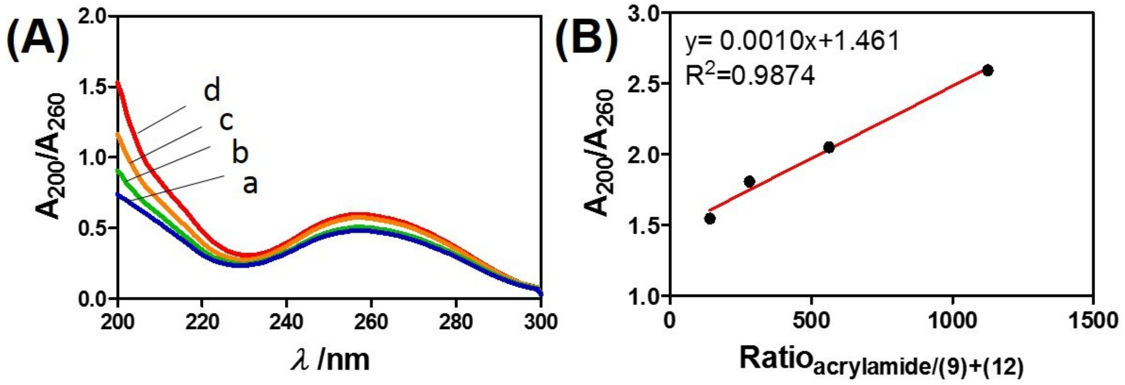

Figure S6. Determination of the ratio between nucleic acid units and acrylamide monomer units on the hairpin (**9**) and tether (**12**) conjugated copolymer P_F. (A) Absorbance spectra corresponding to various concentrations of polyacrylamide (5000 kDa, *ca.* 70343 monomers) in the presence of constant concentration of the acrydite-modified nucleic acid (**9**), 0.75 μM and (**12**), 1.5 μM: (a) 4.5, (b) 8.9, (c) 17.9, and (d) 35.7 nM. (B) Calibration curve between the molar ratio of acrylamide monomer/(**9**)+(**12**) and the ratio of absorbance at wavelength of 200 nm and 260 nm.

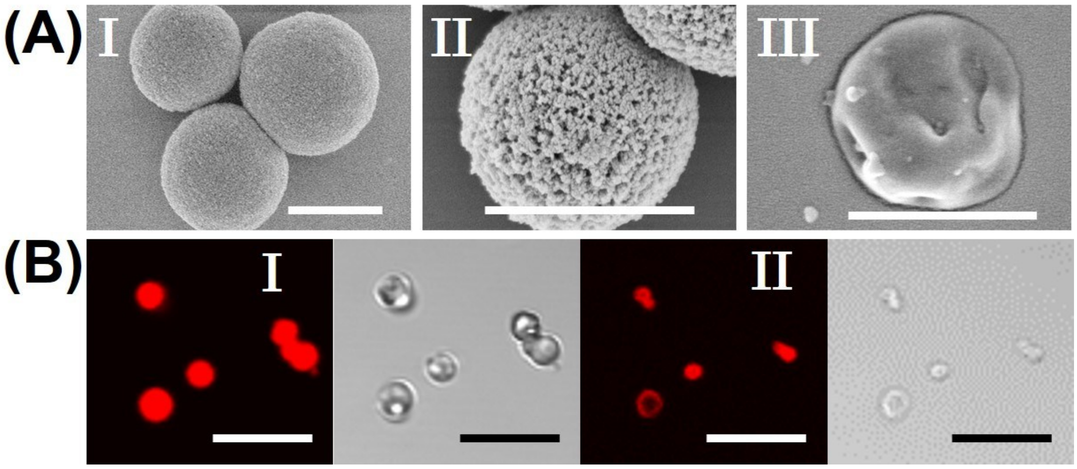

Figure S7. (A) SEM images of uncoated TR-D-loaded $CaCO_3$ microparticls (panel I), cocaine aptamer-bridged hydrogel $CaCO_3$ microparticles (panel II), and TR-D-loaded cocaine-responsive hydrogel microcapsules after the dissolution of the $CaCO_3$ core (panel III). Scale bars, 2 μm (panels I and II) and 1 μm (panel III). (B) Confocal fluorescence microscopy images and bright-field microscopy images of cocaine aptamer-bridged hydrogel microparticles before (panel I) and after (panel II) the dissolution of the core. Scale bar is 10 μm.

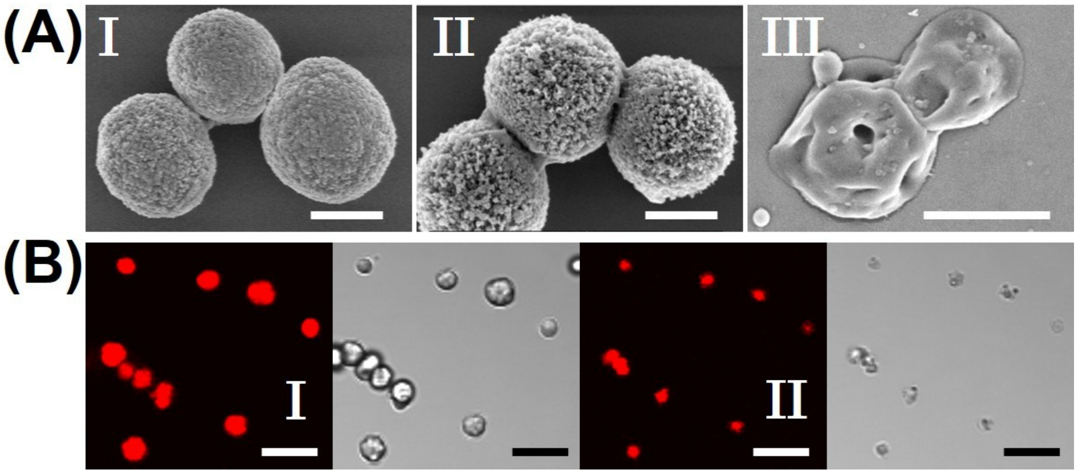

Figure S8. (A) SEM images of uncoated DOX-D-loaded $CaCO_3$ microparticls (panel I), ATP aptamer-bridged hydrogel $CaCO_3$ microparticles (panel II), and DOX-D-loaded ATP-responsive hydrogel microcapsules after the dissolution of the $CaCO_3$ core (panel III). Scale bars, 2 μm (panels I and II) and 1 μm (panel III). (B) Confocal fluorescence microscopy images and bright-field microscopy images of ATP aptamer-bridged hydrogel microparticles before (panel I) and after (panel II) the dissolution of the core. Scale bar is 10 μm.

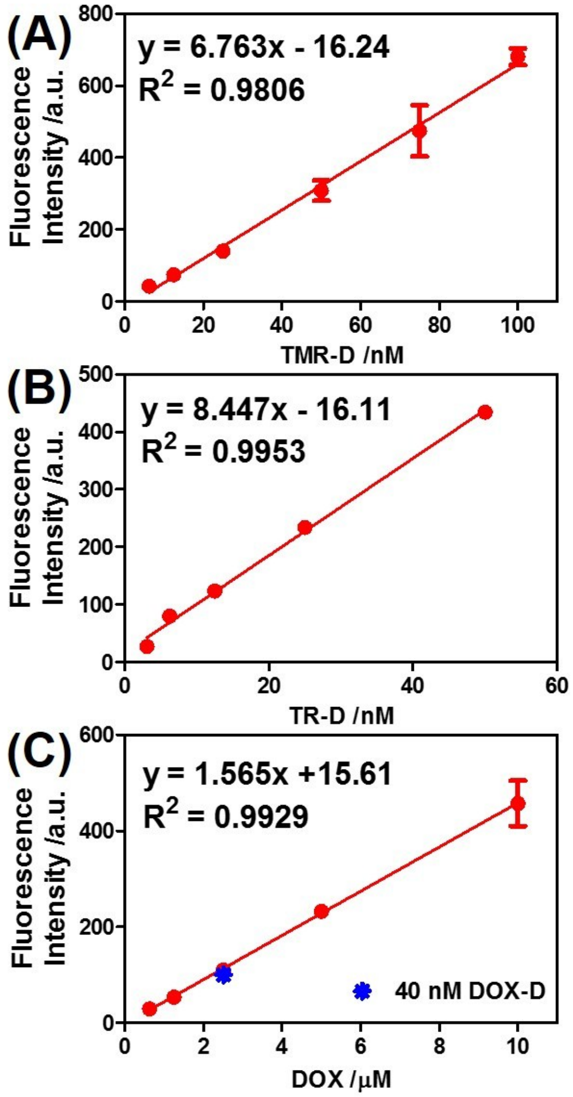

Figure S9. Calibration curves corresponding to (A) tetramethylrhodamine-modified dextran, TMR-D, (B) Texas Red-modified dextran, TR-D, and (C) doxorubicin, DOX. The fluorescence of DOX-D was measured after the acidification that allows for the cleavage of doxorubicin from the dextran.

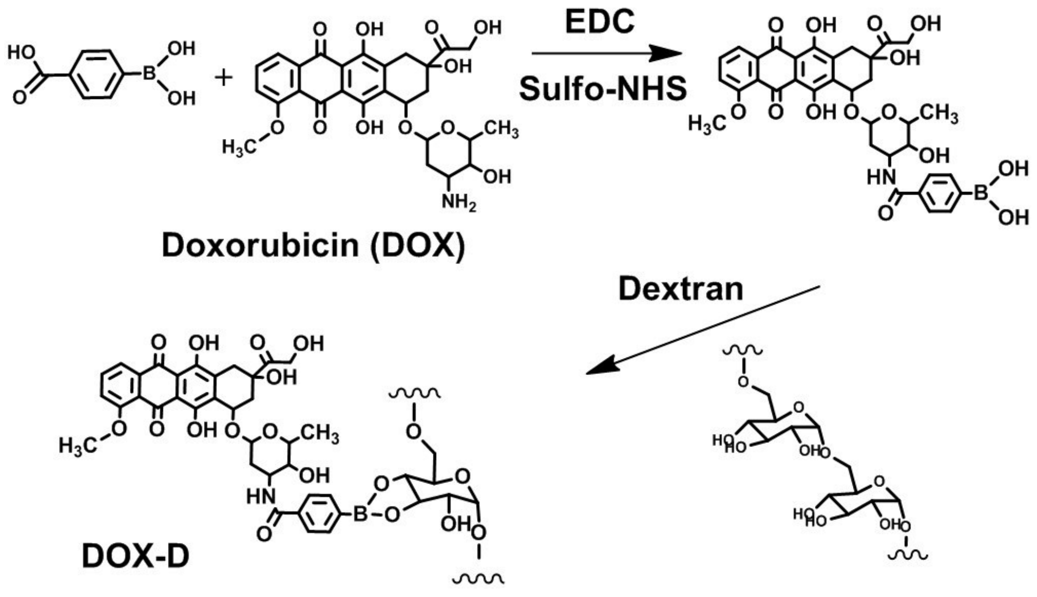

Figure S10. The preparation of doxorubicin-conjugated dextran, DOX-D.

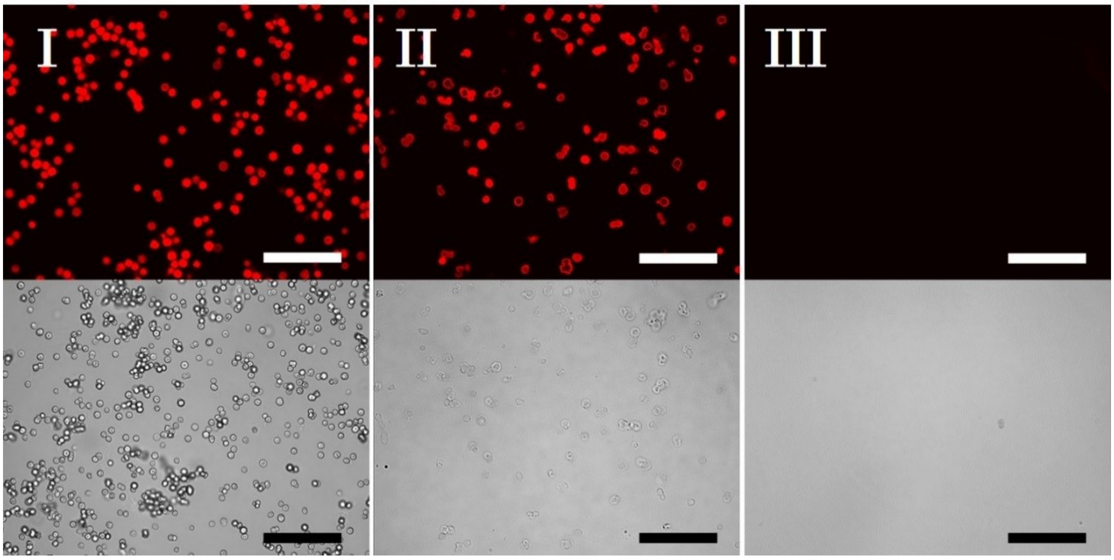

Figure S11. Confocal fluorescence microscopy images and bright-field microscopy images of i-motif-bridged hydrogel microparticles before (panel I) and after (panel II) the dissolution of the core, and subjecting the microcapsules to pH = 5.0 (panel III). Scale bar is 30 μm.

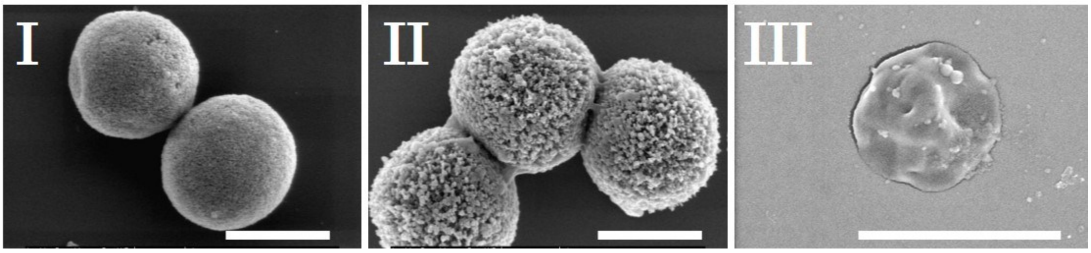

Figure S12. SEM images of uncoated DOX-D-loaded $CaCO_3$ microparticles (panel I), i-motif-bridged hydrogel $CaCO_3$ microparticles (panel II), and DOX-D-loaded pH-responsive hydrogel microcapsules after the dissolution of the $CaCO_3$ core (panel III). Scale bar is 2 μm.

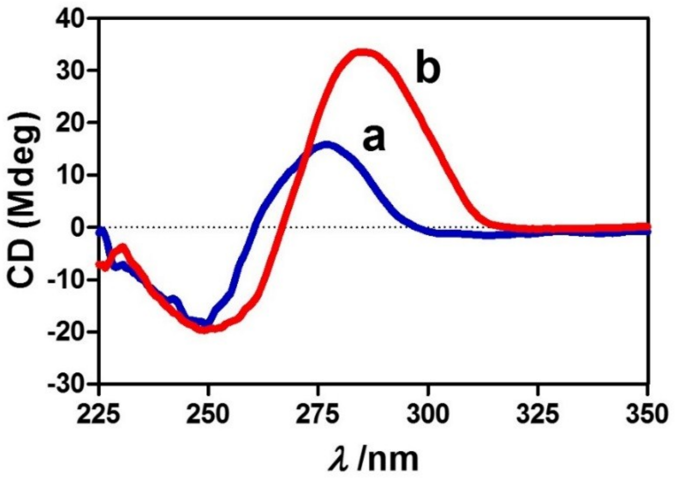

Figure S13. Circular dichroism spectra corresponding to: (a) The pH-responsive acrylamide hydrogel at pH = 7.2. (b) The pH-responsive acrylamide hydrogel at pH = 5.0.

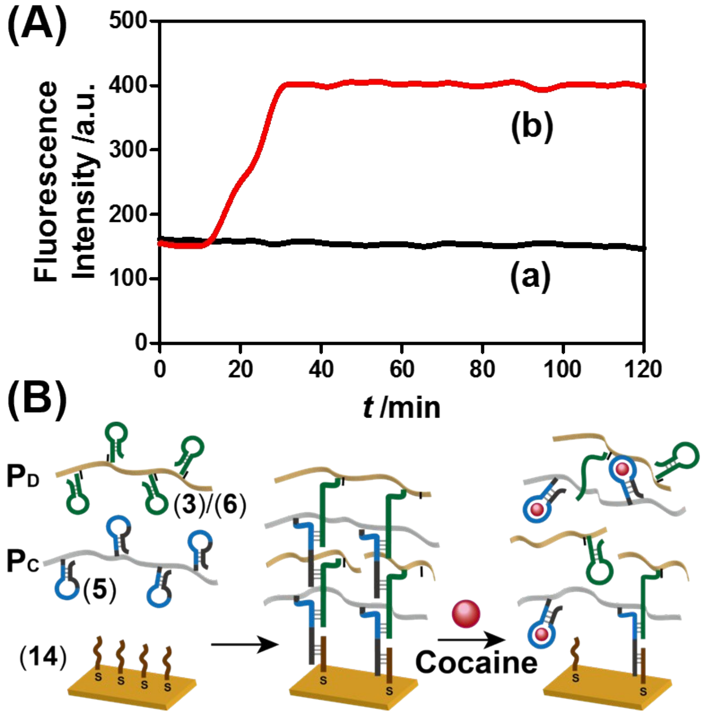

Figure S14. (A) Time-dependent release of TR-D from the cocaine-responsive hydrogel matrix in the absence of cocaine (curve a) and in the presence of 20 mM cocaine (curve b). (B) Schematic illustration of the preparation of the hydrogel membranes on a gold-coated surface and the separation of the hydrogel matrix by the cocaine-trigger. The Young's module of the cocaine-responsive hydrogel corresponded to 123 ± 4 Pa. After treatment of the film with cocaine, 20 mM, the value of the Young's module decreased to 44 ± 18 Pa.

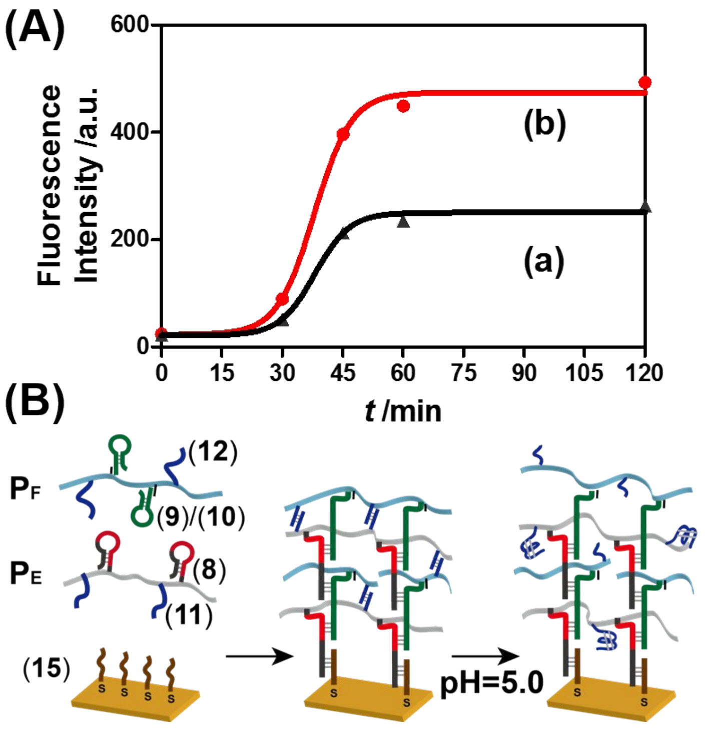

Figure S15. (A) Time-dependent release of TMR-D from the pH-responsive hydrogel matrix subjecting to pH = 7.2 (curve a) and pH = 5.0 (curve b). (B) Schematic illustration of the preparation of the hydrogel membranes on a gold-coated surface and the separation of the hydrogel matrix by the pH-trigger.
